# Regulator of G protein signaling 17 represents a novel target for treating cisplatin induced hearing loss

**DOI:** 10.1038/s41598-021-87387-5

**Published:** 2021-04-14

**Authors:** Asmita Dhukhwa, Raheem F. H. Al Aameri, Sandeep Sheth, Debashree Mukherjea, Leonard Rybak, Vickram Ramkumar

**Affiliations:** 1 Department of Pharmacology, Southern Illinois University School of Medicine, Springfield, IL 62702 USA; 2 Department of Otolaryngology, Southern Illinois University School of Medicine, Springfield, IL 62702 USA; 3 Department of Pharmaceutical Sciences, Larkin University College of Pharmacy, Miami, FL 33169 USA

**Keywords:** Cell biology, Neuroscience, Physiology, Medical research

## Abstract

Regulators of G protein signaling (RGS) accelerate the GTPase activity of G proteins to enable rapid termination of the signals triggered by G protein-coupled receptors (GPCRs). Activation of several GPCRs, including cannabinoid receptor 2 (CB2R) and adenosine A_1_ receptor (A_1_AR), protects against noise and drug-induced ototoxicity. One such drug, cisplatin, an anticancer agent used to treat various solid tumors, produces permanent hearing loss in experimental animals and in a high percentage of cancer patients who undergo treatments. In this study we show that cisplatin induces the expression of the *RGS17* gene and increases the levels of RGS17 protein which contributes to a significant proportion of the hearing loss. Knockdown of *RGS17* suppressed cisplatin-induced hearing loss in male Wistar rats, while overexpression of *RGS17* alone produced hearing loss in vivo. Furthermore, RGS17 and CB2R negatively regulate the expression of each other. These data suggest that RGS17 mediates cisplatin ototoxicity by uncoupling cytoprotective GPCRs from their normal G protein interactions, thereby mitigating the otoprotective contributions of endogenous ligands of these receptors. Thus, RGS17 represents a novel mediator of cisplatin ototoxicity and a potential therapeutic target for treating hearing loss.

## Introduction

Cisplatin is the first FDA-approved platinum drug used for a treatment of solid tumors such as head and neck, bladder, lung, ovarian and testicular cancers^1,2^. Its mechanism of antitumor action involves forming cross-links with DNA purine bases at the N7 position to limit cancer cell replication^3–5^. Despite its wide use clinically since the late 1970s, cisplatin possesses dose limiting adverse side effects such as ototoxicity, neurotoxicity and nephrotoxicity^4,6^.

Cisplatin-induced ototoxicity is progressive and irreversible^7^. It affects almost 13–95% of children treated for neuroblastoma (assessed by CTCAE scale)^8^, even though incidence of ototoxicity was highly dependent on the auditory testing scales used. A similar wide incidence range of ototoxicity has been reported in a recent review of incidences of hearing loss in different countries^9^. Cisplatin-induced hearing loss is associated with increased cochlear cell death resulting from DNA damage, caspase activation, oxidative stress, inflammation and glutamate excitotoxicity^7,10^. These stressors lead to decrease in endocochlear potential, loss of ribbon synapses, loss of outer hair cells and elevations in ABR thresholds^11–13^. Various cytoprotective measures reduce the negative effects of cochlear stressors. These includes the use of antioxidants, anti-inflammatory agents and agents that modulate cell apoptotic pathways^7^. Several GPCRs also exert otoprotection. For example, adenosine A_1_ receptors^14–16^ and cannabinoid receptor 2^12,17^ are expressed in the cochlea, predominantly in the organ of Corti (OC), strial vascularis (SVA) and spiral ganglion neurons (SGN)^12,14,18^. Activation of these GPCRs reduced inflammation, oxidative stress and reduced cisplatin-induced ABR threshold shifts^12,14,19^.

GPCR signaling controls the activation and inactivation of heterotrimeric G proteins. Activation of GPCRs by agonists leads to activation of intracellular effectors which produce second messengers, activate protein kinases, and regulate downstream signaling in order to influence cellular functions^20–22^. In addition to various GPCRs, G proteins such as G_i_, G_o,_ G_s_, G_q,_ G_z_ are expressed in the cochlea^23–25^. These G proteins are present in the OC, SVA and SGN, highlighting the importance of GPCR signaling in cochlea^25–28^. Regulators of G-protein signaling (RGS) promote the inactivation and termination of G protein mediated signaling by facilitating GTP hydrolysis^29^. Transcriptome data analysis of cochleae from control and cisplatin-treated rats performed in our laboratory demonstrate differential expression of RGS proteins. Among these, *RGS17*, a member of RGS-RZ subfamily, which commonly targets GTP bound Gα_i1-3_, Gα_o_, Gα_z_ and Gα_q_ for hydrolysis^30^ was highly induced. RGS17 has been extensively studied in the central nervous system for its role in inhibiting μ-opioid, dopamine and cannabinoid receptors. An interesting function of RGS17 is its role as a potential redox transducer. This is mediated by the interaction of cysteine-rich domain of RGS17 with nitric oxide to release zinc^31^. RGS17 positively influences the protein kinase A-cyclic AMP response element binding protein^32^ pathway by inhibiting Gαi/o signaling to promote proliferation, migration and invasion in cancers.

In this study we test the novel hypothesis that RGS17 is a critical regulator of cochlear function and mediator of cisplatin ototoxicity and works by inhibition of the functions of otoprotective GPCRs. Moreover, we examined the possibility that RGS17 antagonizes the protective actions of the CB2R against cisplatin ototoxicity. Accordingly, inhibition of RGS17 could enhance the protection mediated by these GPCRs by extending the duration of the receptor-G protein interaction.

## Results

### RGS17 is expressed in the rat cochlea

RGS proteins are not widely studied in the auditory system. Among more than 20 RGS proteins, only RGS4 is expressed in SGNs^18^, supporting cells and OC of adult cochlea. Increased level of RGS4 is associated with neuronal defects in PTEN-deficient SGNs^18^. While this manuscript was in the process of being submitted, a published study provided convincing evidence implicating RGS4 in noise-induced hearing loss in rats^33^. Additionally, RGS18 is present in otic vesicles of zebrafish and mouse embryos where it is involved in development of cilia in hair cells of the inner ear^34^. Our study demonstrates the distribution of RGS17 in cochlea using the polyclonal antibody. Validation of this antibody was done in OC-derived UB/OC-1 cells^35^ transiently transfected with *RGS17* overexpressing plasmid, which resulted in increased level of RGS17 protein expression (almost 300% increase) as detected via immunofluorescence and Western blotting (Supplementary Fig. S1A–C). In rodents, RGS17 is predominantly expressed in CNS, specifically in the cerebral cortex, nucleus accumbens and striatum^36,37^ but not in normal liver tissue or hepatocytes^38^. Thus, we characterized the specificity of RGS17 antibody using cerebral cortex as a positive control and liver tissue as a negative control. We observed distinct immune-positive fluorescent labelling of RGS17 in the brain section whereas no significant staining was observed in liver sections (Supplementary Fig. S1D). Furthermore, knockdown and overexpression studies described below show reductions and increases in RGS17 levels, respectively. Our observations confirm the specificity of the antibody we used to detect RGS17 protein is valid and thus we further used it to identify RGS17 in the cochlea.

**Figure 1:**
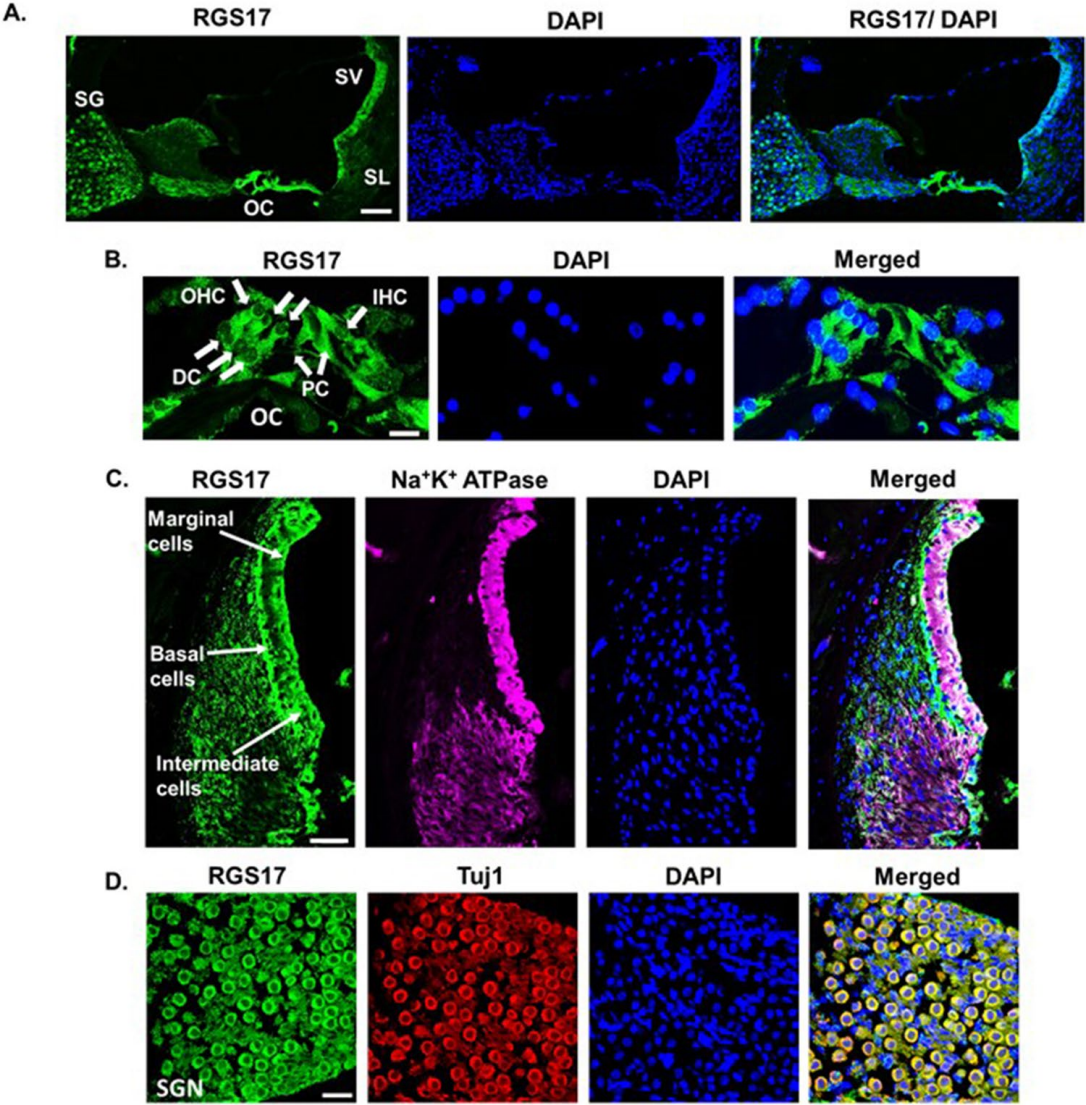
Distribution of RGS17 in the rat cochlea. Naïve male Wistar rats were used for the collection of cochleae. Collected cochleae were fixed with 4% PFA, cryo-frozen and processed for mid-modiolar sectioning using cryostats. Mid-modiolar section was immunolabeled with RGS17 (green) and DAPI (blue). (**A**) Significant immunolabelling of RGS17 observed in the rat cochlea. (**B**) Expression of *RGS17* was observed in OHCs, IHC, supporting cells: DCs, IPC and OPC. (**C**) Co-immunolabelling with Na^+ ^K^+^ ATPase (magenta) shows expression of *RGS17* in the lateral wall of the cochlea. Positive immunofluorescence was seen in basal, marginal, intermediate cells and SL. (**D**) Co-staining with Tuj1 (red) shows *RGS17* expression in cochlear neuronal cells, SGNs (Scale 50 μm).

The mid-modiolar sections of the cochlea from male Wistar rats showed distinct RGS17 immunoreactivity. Positive immunolabeling was observed in OC, SGNs and SV (Fig. 1A). In OC, both sensory and non-sensory cells—outer hair cells (OHCs), inner hair cells (IHCs), inner pillar cells (IPCs), outer pillar cells (OPCs) and Deiters cells (DCs) expressed RGS17 (Fig. 1B). These mid-modiolar sections were also co-labeled with antibodies against Na^+^/K^+^/ATPase and RGS17 to investigate the expression of the latter in the lateral wall of the cochlea. Na^+^/K^+^/ATPase was used as a marker for the lateral wall as it is expressed in strial cells and fibrocytes of spiral ligament^11,39^. Intense RGS17 immunolabelling was observed in the basal cells of SVA, compared to marginal cells, and the least fluorescent intensity was found in intermediate cells (Fig. 1C). In addition, co-staining of RGS17 with Tuj1, an antibody for class III β-tubulin that labels both type of SGNs, demonstrated the expression of RGS17 in Type I and Type II SGNs (Fig. 1D). Taken together, these data strongly support the presence and localization of RGS17 in the rat cochlea.

### Cisplatin induces RGS17 expression

**Figure 2:**
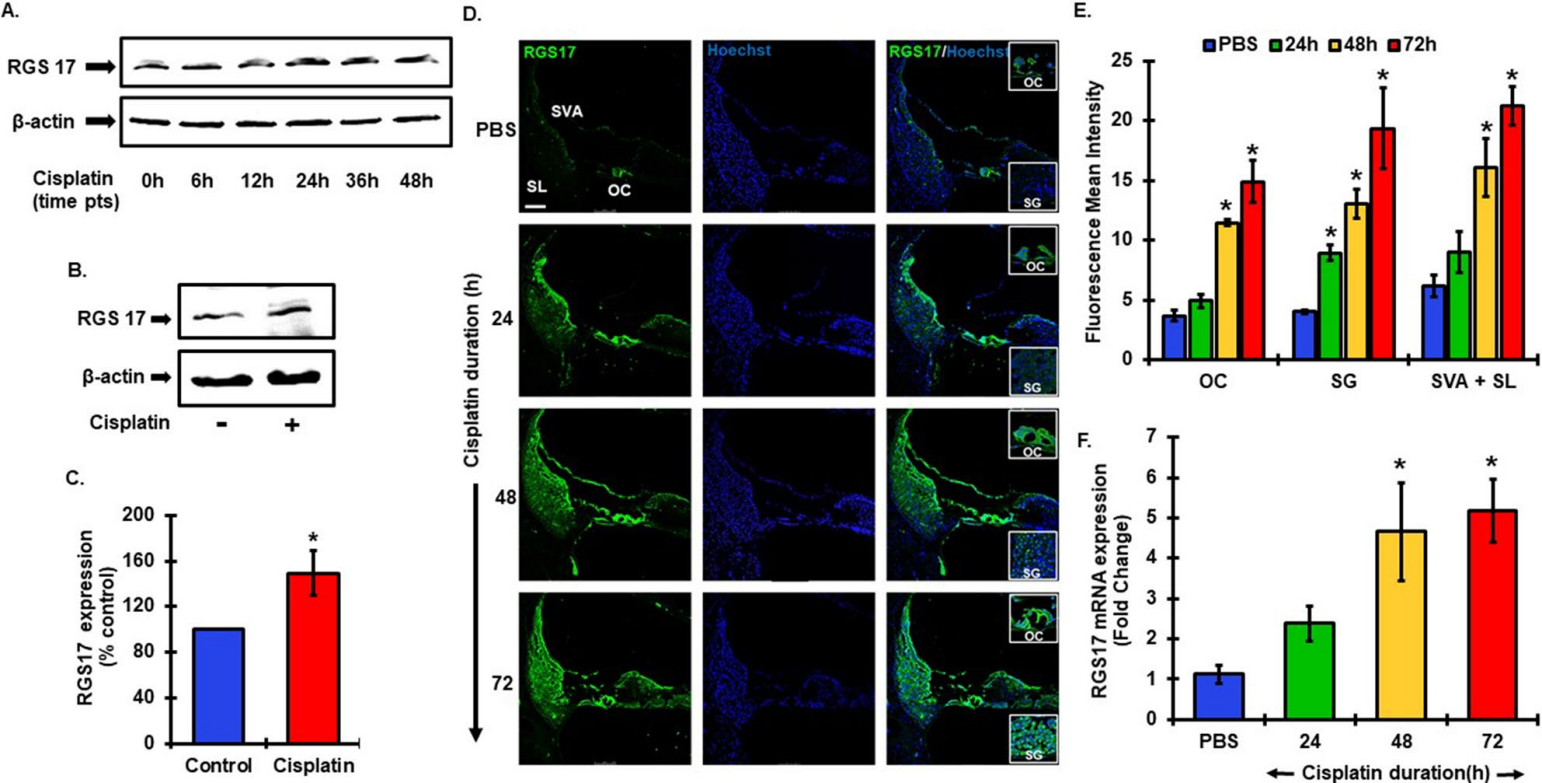
Cisplatin administration increased *RGS17* expression and protein levels in both in vitro and in vivo models. (**A**) UB/OC1 cells were treated with cisplatin (20 μM) for 6, 12, 24, 36 and 48 h. Cell lysates were prepared for western blot analysis which showed increase in RGS17 expression post cisplatin treatment. (**B**) Western blotting result showed increased protein level expression of RGS17 after 24 h of cisplatin (20 μM) treatment. (**C**) Graphical representation of western blot analysis from (**B**) showing RGS17 expression significantly increased after 24 h of cisplatin treatment. Data represents mean ± SEM, *p < 0.05, N = 4, independent t-test. (**D**) Male Wistar rats were treated with cisplatin (11 mg/kg, i.p) or sterile PBS (1 ml, i.p) for 24 h, 48 h and 72 h. Cochleae from treated animals were fixed with 4% PFA and processed for mid-modiolar sections. Cochlear sections were immunolabeled with RGS17 (green) and Hoechst (blue). Cisplatin treated animals showed notably higher level of fluorescence indicating increased the levels of RGS17 as compared to PBS treated animals at 24, 48 and 72 h post treatment. (**E**) Bar graph representing the mean fluorescence intensity of RGS17 immunostaining (refer (**D**)) in different region of the cochlea: OC, SG and lateral wall (SL + SVA). Data represents mean ± SEM, *p < 0.05, N = 4, two-way anova. (**F**) Cochleae were also collected for RNA extraction for RT-qPCR analysis. Cisplatin treatment increased *RGS17* l expression at all time points tested and it was significantly elevated at 48 h and 72 h. Data indicate mean fold change in the mRNA levels ± SEM, N ≥ 3, *p < 0.01 vs. PBS, one-way ANOVA.

Our laboratory has previously shown that agonist activation of GPCRs such as A_1_AR and CB2R protect against cisplatin-induced hearing loss^12,14^. Furthermore, we showed that knock down of CB2R in the cochlea aggravated the cisplatin-induced ototoxicity, as evidenced by increased ABR threshold shifts^12^. These data suggest endogenous “cytoprotective” GPCRs like CB2R could protect against cisplatin ototoxicity following activation by endocannabinoids or by administration of exogenous agonists of this receptor. To determine whether cisplatin could inhibit this endogenous defense system, we examined whether this agent could induce the expression of *RGS17* in the cochlea. In effect, induction of *RGS17* is expected to antagonize GPCR (such as CB2R) -G protein interaction and function by accelerating hydrolysis of bound GTP to GDP. We tested this hypothesis in vitro in UB/OC-1 cells, an organ of Corti-derived clonal cell line, which were treated with cisplatin (20 μM) over different time points, 6 h, 12 h, 24 h, 36 h and 48 h. Cisplatin produced time-dependent increases in RGS17 protein level which peaked by 24 h (149% ± 20) and remained relatively stable at 36 and 48 h post treatment (Fig. 2A–C). Similar observations were reported using RT q-PCR and immunofluorescence (data not shown). For in vivo studies, we treated male Wistar rats with cisplatin for 24 h, 48 h and 72 h. Cochleae collected from treated animals along with control (PBS-treated) animals were collected for either RNA extraction or fixed for cryo-sectioning. Cisplatin treated animals showed increased RGS17 immunoreactivity as observed in mid-modiolar sections. Immunostaining showed increasing fluorescence intensities from 24 h, 48 h and to 72 h (Fig. 2D). While the intensity of the RGS17 image in the vehicle looks weak, it was captured at a lower intensity (than that of Fig. 1) to better depict the relatively high RGS17 fluorescence in the cisplatin group. We have presented the vehicle/cisplatin comparison images at 72 h in Supplementary Fig. S2, which were captured at comparable increasing intensities. The fluorescence intensities of RGS17 immunolabeling were increased in all three regions (OC, SGs and lateral wall) of the cochlea after cisplatin treatment (Fig. 2E). RGS17 immunoreactivity was significantly higher in OC (4.9 ± 0.5, 11.5 ± 0.3 and 14.9 ± 1.7 intensity units) and the lateral wall, SVA + SL (9.0 ± 1.7,  16.1 ± 2.4 and 21.3 ± 1.6) post 24, 48 and 72 h cisplatin treatment, respectively. RGS17 fluorescence intensities was also significantly increased in SG: 8.9 ± 0.6, 13.0 ± 1.2 and 19.4 ± 3.4 at 24, 48, and 72 h respectively. Similarly, RT-qPCR analysis of RNA obtained from the cochleae showed cisplatin significantly increased *RGS17* expression at 24, 48 and 72 h by 2.1 ± 0.4, 4.1 ± 1.1 and 4.6 ± 0.7 fold, respectively (Fig. 2F). These data suggest that the *RGS17* gene is susceptible to regulation by cisplatin in the cochlea.

### Functional role of RGS17 in hearing

**Figure 3:**
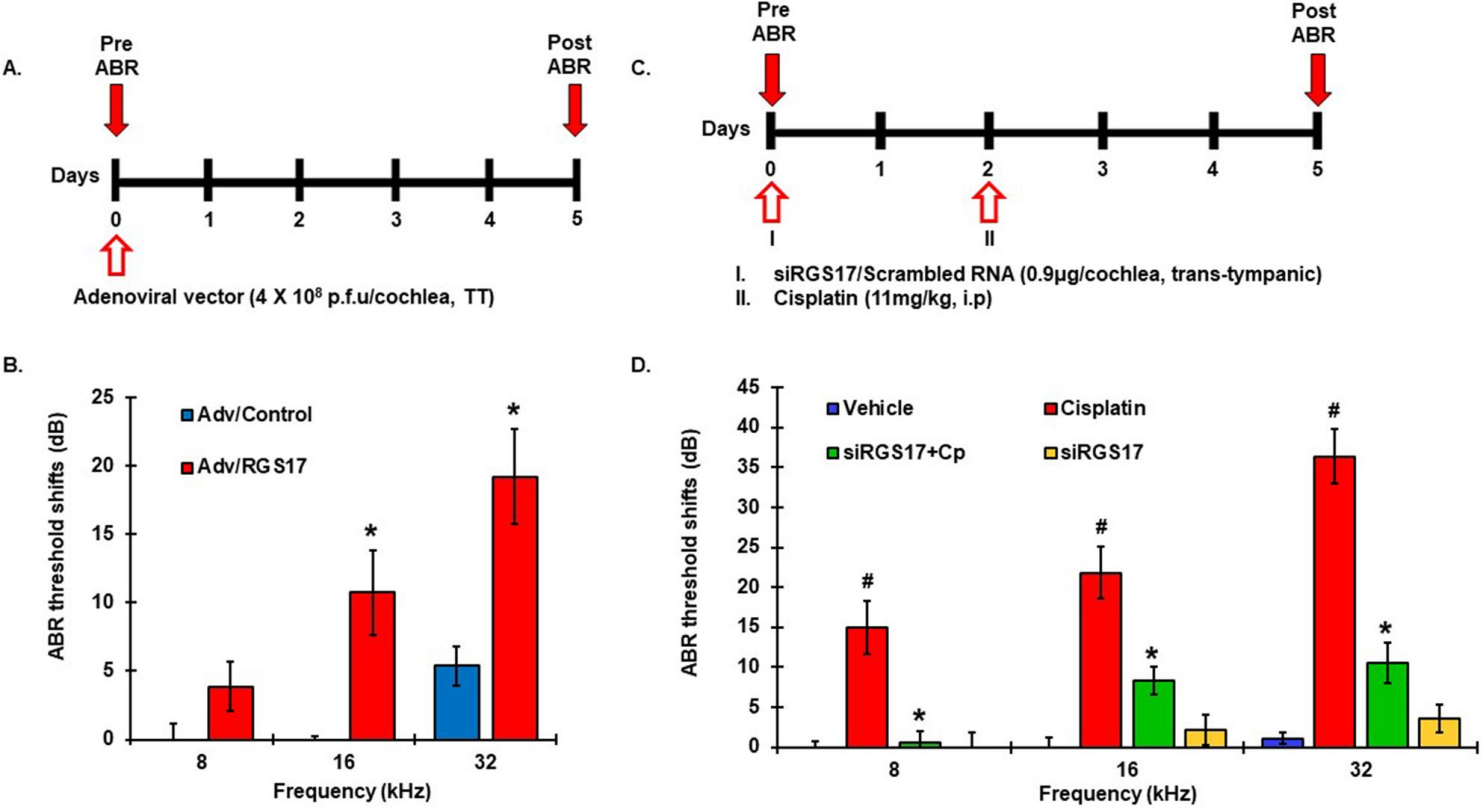
*RGS17* overexpression compromised hearing ability whereas knocking down of *RGS17* rescued hearing in rats. (**A**) Schematic diagram of the experimental protocol that illustrates the dosage and routes of administration of different treatment. Pre-treatment ABRs were measured prior to any drug administration on naïve male Wistar rats. Rats were administered either control adenoviral vector or adenoviral vector overexpressing *RGS17* into ears via trans-tympanic delivery and post-treatment ABRs were assessed after 5 days. (**B**) Adenoviral vector overexpressing *RGS17*  in one ear showed significant increase in ABR threshold shifts than the control ear at 16 and 32 kHz. (**C**) Schematic diagram showing siRGS17 or scrambled RNA trans-tympanically administered immediately after pre-ABRs. Intraperitoneal administration of cisplatin was performed 2 days later for cisplatin-treated groups. Post-treatment ABRs were determined 3 days after cisplatin treatment. (**D**) Post ABR results showed significant elevations in threshold shifts with cisplatin at 8, 16 and 32 kHz. siRGS17 pretreatment significantly attenuated cisplatin-induced elevation in ABR threshold shifts. Data indicate mean ± SEM. ^#^p < 0.01 versus. vehicle, *p < 0.01 versus cisplatin, N  = 12, two-way ANOVA. *TT* trans-tympanic, *i.p* Intraperitoneal.

To elucidate the role of RGS17 in hearing, we overexpressed *RGS17* into the cochlea by trans-tympanic injection of an adenoviral vector tagged with m-cherry (m-cherry-Adv-*RG17*) at a concentration of 4 × 10^8^ plaque forming units/cochlea. The contralateral ears were administered the same concentration of adenoviral vector without *RGS17* and served as the vehicle-controls. Auditory brain responses (ABRs) were assessed five days after the adenoviral vector treatment (Fig. 3A). Adenoviral infection produced a significant increase (~ threefold) in the expression of *RGS17* in the cochlea by 5 days, especially in the SVA and SG (Supplementary Fig. S3A,B). This produced significant elevations in ABR thresholds of 10.8 ± 3.1 dB (at 16 kHz) and 19.2 ± 3.5 dB (at 32 kHz) when compared to the vehicle control ears (Fig. 3B) but not at 8 kHz frequency.

To investigate the role of RGS17 in cisplatin-induced hearing loss model, we administered siRNA against *RGS17* via trans-tympanic route (0.9 μg) into the middle ear of male Wistar rats, two days prior to cisplatin treatment. The control group received a scrambled nucleotide sequence. Cisplatin (11 mg/kg) or vehicle were then administered by intraperitoneal administration over a 30 min period. Pre-ABRs were performed before trans-tympanic administration and post-ABRs were recorded 3 days after cisplatin treatment (Fig. 3C). Efficient knockdown of RGS17 (70%) was observed by siRNA when compared to the scrambled sequence (Supplementary Fig. S3C). Post-treatment ABRs assessed 3 days following cisplatin treatment were 15.0 ± 3.3, 21.8 ± 3.3 and 36.4 ± 3.4 dB at 8, 16 and 32 kHz, respectively, compared to vehicle-treated controls. *RGS17* siRNA significantly decreased ABR threshold shifts as compared to cisplatin alone groups to 0.6 ± 1.5, 8.3 ± 1.7 and 10.6 ± 2.5 at 8, 16 and 32 kHz, respectively (Fig. 3D) and actual threshold values (Supplementary Fig. S3D).

### RGS17 is implicated in synaptopathy in cochlea

**Figure 4:**
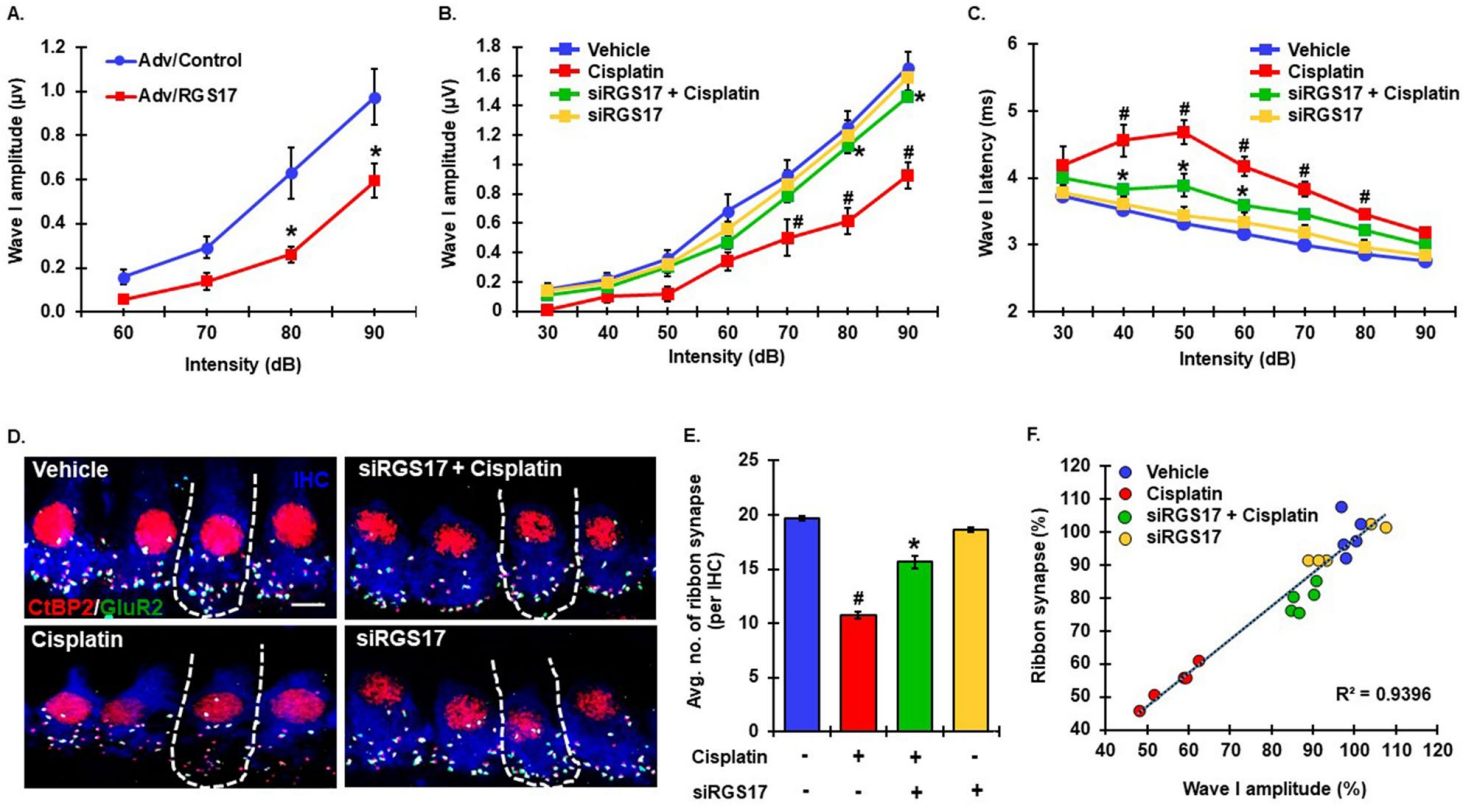
RGS17 regulates cochlear synaptopathy. (**A**) Male Wistar rats were administered adenoviral vector overexpressing *RGS17* or empty viral vector by the trans-tympanic route. Post ABRs were performed 5 days after the treatment. Wave I amplitudes were measured manually at 32 kHz for 60, 70, 80 and 90-dB intensities. *RGS17* overexpression significantly decreased wave I amplitudes at 80 and 90-dB. Data indicates mean ± SEM, *p < 0.05 versus adenovirus/control, N = 10, two-way ANOVA. (**B**) Naïve male Wistar rats were treated with siRGS17 (0.9 μg/ear) 2 days prior to cisplatin administration. Post-treatment ABRs were performed 3 days after cisplatin exposure and wave I amplitude was measured at 32 kHz for 30–90 dB intensities. Rats were divided into 4 treatment groups, vehicle (blue line), cisplatin (red line), siRGS17 pretreatment + cisplatin (green line) and siRGS17 alone (yellow line) alone. Cisplatin produced a significant decrease in wave I amplitude at 70, 80 and 90-dB intensities whereas knock down of *RGS17* prior to cisplatin significantly abrogated this response at 80 and 90-dB, compared to cisplatin treated rats. Data represents mean ± SEM, ^#^p < 0.01 versus vehicle, *p < 0.01 versus cisplatin, N  = 12, two-way ANOVA. (**C**) In the same group of animals, cisplatin treatment significantly increased Wave I latencies at 32 kHz for 40–80 dB intensities but pretreatment of siRGS17 significantly decreased wave I latencies at 40, 50 and 60-dB. Data represents mean ± SEM, ^#^p < 0.05 versus vehicle, *p < 0.05 versus cisplatin, N  = 12, two-way ANOVA. (**D**) The cochleae collected from rats after ABRs were micro-dissected and co-stained with antibody against Myo7a (blue), CtBP2 (red) and GluR2 (green) for immunofluorescence assays. Representative images are taken at the level of IHCs from the basal region of the cochleae. Colocalization of CtBP2 (red) and GluR2 (green) on a background of Myo7a (blue) merge together to give bright white puncta which represents functional ribbon synapse at IHCs. Cisplatin treatment decreased the abundance of white punctae but pretreatment of siRGS17 preserved the number of white punctae. Both vehicle and siRGS17 treated cochleae displayed normal number of white puncta. (**E**) Presence of white puncta was manually counted and represented in the graph as the the average number of functional ribbon synapses per IHC. Cisplatin significantly decreased the average number of functional synapses at IHC as compared to siRGS17 pretreated cochleae. Data plotted represents average mean of functional synapse ± SEM (N ≥ 4). #p < 0.05 compared to vehicle, *p < 0.05 compared to cisplatin, N = 4, two way ANOVA (**F**) ABR Wave I amplitudes (at 90 dB) collected from (**B**) and synaptic count from (**D**) were normalized with respect to the mean values in their vehicle controls. Correlation graph was plotted for these two parameters which shows high correlation between them with R^2^ = 0.94. Data represents N = 4.

We next explored whether RGS17 is also implicated in cochlear synaptopathy. Overexpression of *RGS17* in the cochlea led to significant reductions in wave 1 amplitudes observed at 80 dB by 58.7% (from 0.63 ± 0.12 to 0.26 ± 0.04 µV) and by 70.0% (from 0.98 ± 0.13 to 0.59 ± 0.08 µV) at 90 dB. No significant changes were observed at 60 and 70 dB intensities. These values were reduced by 63.5% (0.16 ± 0.03 and 0.06 ± 0.02 µV) at 60 dB and 52.3% (0.29 ± 0.05 and 0.14 ± 0.04 µV) at 70 dB by *RGS17* overexpression (Fig. 4A). Similar to the *RGS17* vector, cisplatin significantly reduced wave 1 amplitudes, while trans-tympanic administration of *RGS17* siRNA, which did not affect wave 1 amplitudes by itself, reduced the magnitude of the cisplatin’s effects at 80 and 90 dB (Fig. 4B). The amplitudes in the vehicle at 60, 70, 80 and 90 dB were 0.68 ± 0.12, 0.93 ± 0.10, 1.23 ± 0.11 and 1.66 ± 0.11 µV, respectively. The amplitudes in the cisplatin group at 60, 70, 80 and 90 dB were 0.34 ± 0.06, 0.50 ± 0.12, 0.62 ± 0.09 and 0.93 ± 0.09 µV, respectively. *siRGS17* pretreatment with cisplatin preserved wave I amplitudes. Wave 1 amplitudes measured at 60, 70, 80 and 90 dB in this group were 0.47 ± 0.05, 0.79 ± 0.04, 1.13 ± 0.06, and 1.46 ± 0.02 µV, respectively. siRGS17, administered alone, did not significantly alter wave 1 amplitudes measured at 60, 70, 80 and 90 dB. These values were 0.56 ± 0.06, 0.86 ± 0.10, 1.20 ± 0.10 and 1.59 ± 0.09 µV, respectively.

Peak latency represents the time interval between the onset of acoustic stimuli and the initiation/occurrence of peak of the corresponding wave. Wave I latency signifies the time required for neural impulses to conduct the information from IHC to cochlear nerve. We found that cisplatin significantly delayed the response latencies compared to control animals across a broad range of sound intensities (40–80 dB): 4.56 ± 0.23 ms, 4.68 ± 0.18 ms, 4.17 ± 0.13 ms, 3.83 ± 0.11 ms, 3.45 ± 0.08 ms, respectively, whereas siRGS17 pretreatment significantly shortened the peak latencies at 40, 50 and 60 dB by at least 14%: 3.83 ± 0.08 ms, 3.89 ± 0.17 ms, 3.59 ± 0.07 ms respectively (Fig. 4C).

Functional characteristics of wave I go in parallel with the integrity of synapse at the junction of IHC and afferent nerves from SGN I neurons^40,41^. Previously, we have shown that cisplatin decrease cochlear ribbon synapses, whereas activation of CB2 receptors prevented the loss of synapses to promote normal hearing^12^. We quantified the number of C-terminal binding protein 2 (CtBP2)-immuno-positive puncta as an indicator for ribbon synapses at IHC and post synaptic glutamate receptor, GluR2, -immuno-positive puncta as a post synaptic marker at the afferent nerve. The functional synapse is reported as a puncta/dot co-stained with both the markers. Cisplatin treatment reduced the number of functional ribbon synapses (as marked by decreased number of bright white puncta shown by the combination of three colors: red (CtBP2), green (GluR2) and blue in region of IHC (Fig. 4D). Rats pretreated with *RGS17* siRNA maintained the same number of functional synapses (Fig. 4D,E). Cisplatin significantly reduced the average number of functional ribbon synapses to 10.7 ± 0.3, as compared to 19.7 ± 0.20 for control. However, *siRGS17* significantly attenuated this loss of synapses (observed with cisplatin) to 15.7 ± 0.6 (Fig. 4E). The number of functional synapse were counted manually and results were plotted in the graph. A regression plot analysis of wave I amplitude versus ribbon synapse counts showed that these two variables are positively related across the different treatment groups with correlation coefficient, R2 = 0.94 and slope (m) = 1.01 (Fig. 4F). This strong correlation indicates that the reduction in wave 1 amplitude and functional synapses with cisplatin treatment and the protection afforded by *RGS17* knockdown. Our data suggests *RGS17* could be a potential therapeutic target for alleviating synaptopathy associated with cisplatin ototoxicity.

### Knockdown of RGS17 protects against cisplatin-induced apoptosis in cochlea

**Figure 5:**
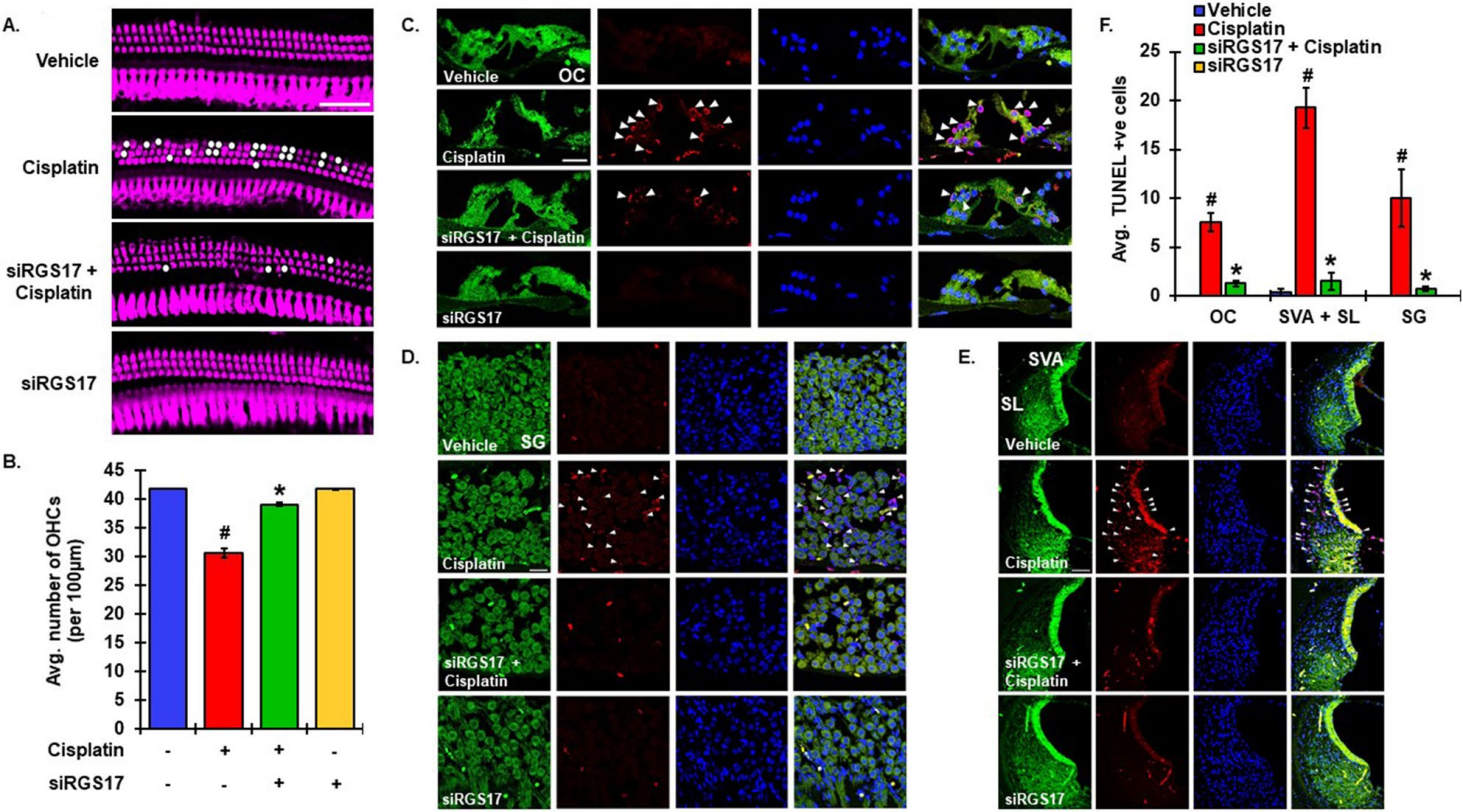
Knockdown of RGS17 inhibits cisplatin-mediated apoptosis in cochlea. (**A**) Male Wistar rats were pretreated with siRGS17 (0.9 μg/ear), followed by cisplatin (11 mg/kg, ip) 48 h later. Rats were sacrificed after 3 days. Cochleae were harvested, fixed, decalcified and micro-dissected and whole mount sections from the basal turns were stained with myosin VIIa (magenta). Representative images showed higher degree of damage to OHCs after cisplatin treatment, compared to siRGS17 pre-treated cochleae. Scale bar represents 25 μm. (**B**) Graphical representation of OHC loss showed significant decrease in number of OHCs in siRGS17 treated animals as compared to cisplatin exposed animals. Bar graphs show the average number of OHCs per 100 µm area. (**C**) Mid-modiolar sections were used for TUNEL staining (red) along with phalloidin immunostaining (green). TUNEL-positive cells as marked by white arrows were observed to be higher in both hair cells and supporting cells of cisplatin treated animals as compared to siRGS17 pretreated cochleae. (**D**) In SG neurons as well as in SVA (**E**), cisplatin produced greater number of TUNEL-positive cells and no distinct TUNEL-positive staining was observed in cochleae treated with siRGS17. Scale bar represents 50 µm (SVA) and 25uM (OC and SG). (**F**) Bar graph from TUNEL assay presented in (**C,D,E**) showed significantly higher number of TUNEL positive cells in animals after cisplatin exposure whereas it significantly decreased in animals pretreated with siRGS17. Data are presented as mean ± SEM. ^#^p < 0.01 versus vehicle, *p < 0.01 versus cisplatin, N ≥ 4, one-way ANOVA (**B**) and two-way ANOVA (**F**).

Cisplatin is known to produce apoptosis primarily via ROS generation or by directly intercalating into DNA in cochlea cells^3,4^. Various studies have shown TUNEL positive cells in OHCs, SVA and SGNs^11,42,43^ which were more pronounced in basal turn of the cochlea^11,12^. To test if RGS17 is involved in OHC loss, the basal turns from whole-mount preparations of cochleae collected from previous treatment groups (described in Fig. 3) were stained for myosin VIIa (magenta) and loss in OHCs was indicated as white dots (Fig. 5A). Fluorescence imaging of stained section 30.5 ± 0.8 OHCs (per high power field) remaining in cisplatin-treated group compared to 39.1 ± 0.2 in siRGS17 pretreated group (Fig. 5B). Administration of vehicle or siRGS17 alone did not produce any significant change in the number of OHCs (Fig. 5A,B). No significant TUNEL-positive cells were observed in animals pretreated with siRGS17 before cisplatin administration. Both vehicle control and siRGS17-treated animals did not show any significant TUNEL staining (Fig. 5C–E). Cochleae collected from rats treated with cisplatin demonstrated an average of 7.0 ± 0.9, 19.3 ± 2.1 and 10.0 ± 2.9 TUNEL positive cells in OC, lateral wall including spiral ligament and SVA, and SGNs, respectively (Fig. 5F). Trans-tympanic injection of siRGS17 two days prior to cisplatin administration significantly reduced average number of TUNEL positive cells to 1.3 ± 0.2, 1.5 ± 0.8 and 0.8 ± 0.3 in OC, lateral wall and SGNs, respectively (Fig. 5F). These data are in agreement with in vitro data in UB/OC-1 cells showing that the RGS17 inhibitor, celastrol, protects against cisplatin-induced cell viability (unpublished data).

### Overexpression of *RGS17* disrupts STAT1/STAT3 balance

**Figure 6:**
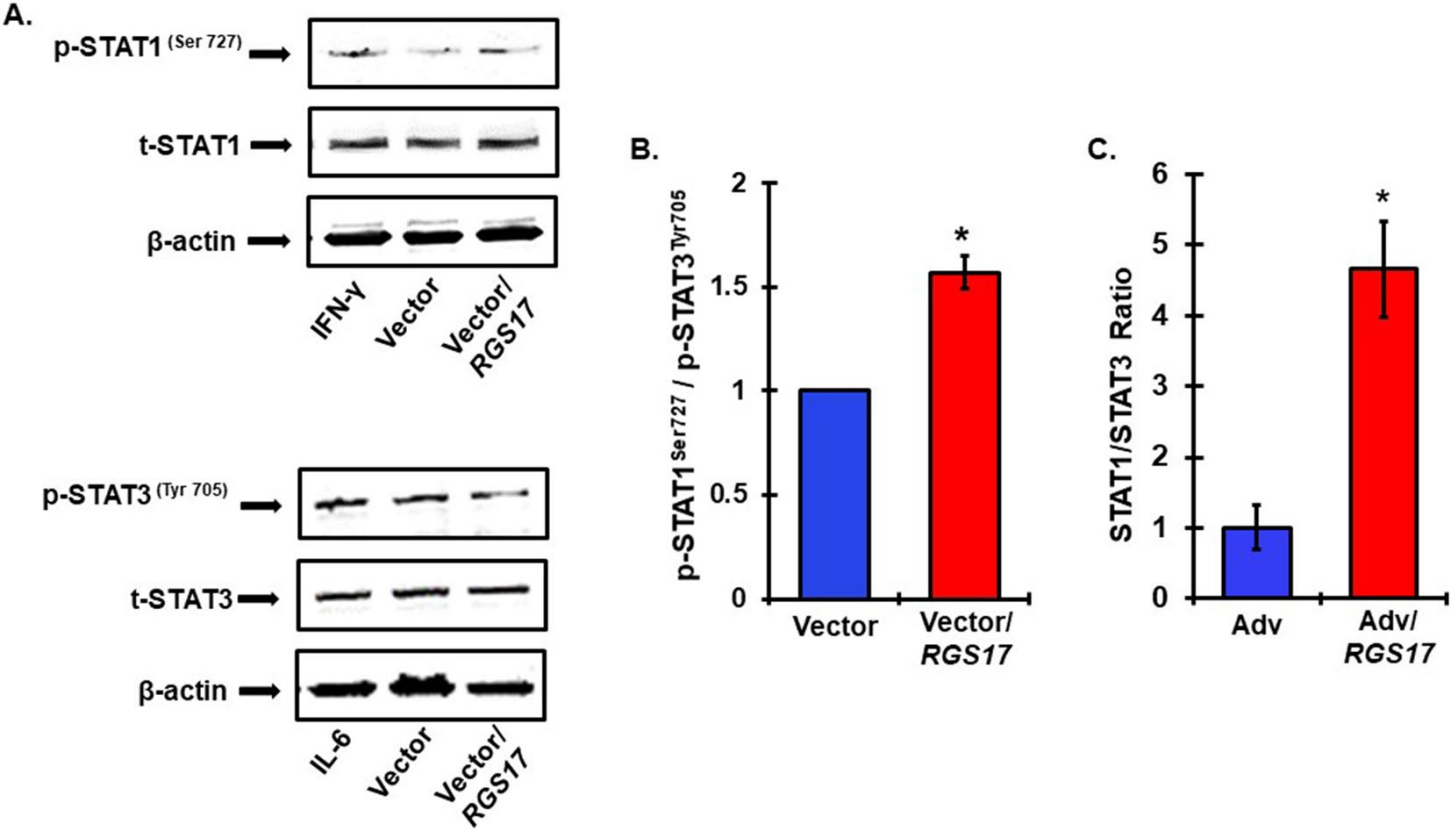
*RGS17* increases STAT1/STAT3 ratio in vivo and in vitro. (**A**) UB/OC1 cells were transiently transfected with control vector or *RGS17* plasmid for 72 h. Cells were treated for IFN-γ and IL-6 for 45 min as positive control for STAT1 and STAT3 respectively. Cell lysates were prepared for western blot analyses of Ser^727^ p-STAT1, Tyr^706^ p-STAT3, t-STAT1 and t-STAT3 for normalization and β-actin for loading control. *RGS17* overexpression increased p-STAT1 activation and decreased p-STAT3 activation (Below). (**B**) Graphical representation showing *RGS17* overexpression increased the STAT1/STAT3 ratio. Data presented as the mean ± SEM. (N = 5). Asterisks *p < 0.01 versus vector, t-test. (**C**) RNA was collected from the whole cochleae of rats treated with empty adenoviral vector or adenoviral vector with *RGS17* transgene. Expression of STAT1 and STAT3 was assessed via real-time qPCR analysis *RGS17* overexpression in rat cochlea significantly increased STAT1/STAT3 ratio. Data indicate mean ratio computed from observed fold change in the mRNA levels ± SEM (N = 4). *p < 0.05 versus Adv and analyzed by t-test.

STAT1 and STAT3 show differential effects on cisplatin-induced apoptosis. A non-selective inhibitor of STAT1, epigallocatechin gallate (EGCG), protected against cisplatin-induced cell death^11^. Both EGCG and capsaicin inhibit the pro-inflammatory pathway and promote cell survival by increasing the STAT3/STAT1 ratio^11,44^. Since, knockdown of *RGS17* ameliorated cisplatin-induced apoptosis in cochlea (see above), we investigated its role in affecting the STAT1/STAT3 balance. We transiently over-expressed *RGS17* in UB/OC-1 cells using a plasmid vector. Western blots performed after the transfection showed an increase activation of p-STAT1 but decreased p-STAT3 activation as compared to the empty vector transfected cells (Fig. 6A). The effects of interferon gamma (IFN-γ) and interleukin-6 (IL-6), positive controls for STAT1 and STAT3 activation, respectively, were tested. In UB/OC-1 cultures, RGS17 significantly increased the ratio of p-STAT1/p-STAT3 to 1.6 ± 0.1-fold, compared to empty vector control (Fig. 6B). In vivo study indicate increased mRNA levels of *STAT1* and reduced levels of *STAT3* in the cochlea infected with *RGS17* adenoviral vector compared to empty vector controls. Cochleae collected from rats overexpressing *RGS17* (refer to Fig. 3B) demonstrated a significantly higher ratio of *STAT1*/*STAT3* (4.6 ± 0.7-fold), compared to control cochleae (Fig. 6C).Thus, overexpression of *RGS17* tilted the ratio towards greater dominance of STAT1 over STAT3 signaling in the cultured cells and the cochlea. We propose that RGS17 could therefore alter the cellular environment of the cochlea (via STAT1/STAT3 ratio), which renders it more susceptible to hearing loss. Interestingly, ChIP-seq data indicate that STAT1 binding sites are present in the *RGS17* promoter^45^. Furthermore, data from our lab indicate that inhibition of STAT1 reduced the expression of *RGS17* (unpublished data).

### Role of RGS17 in redox signaling in the cochlea

**Figure 7:**
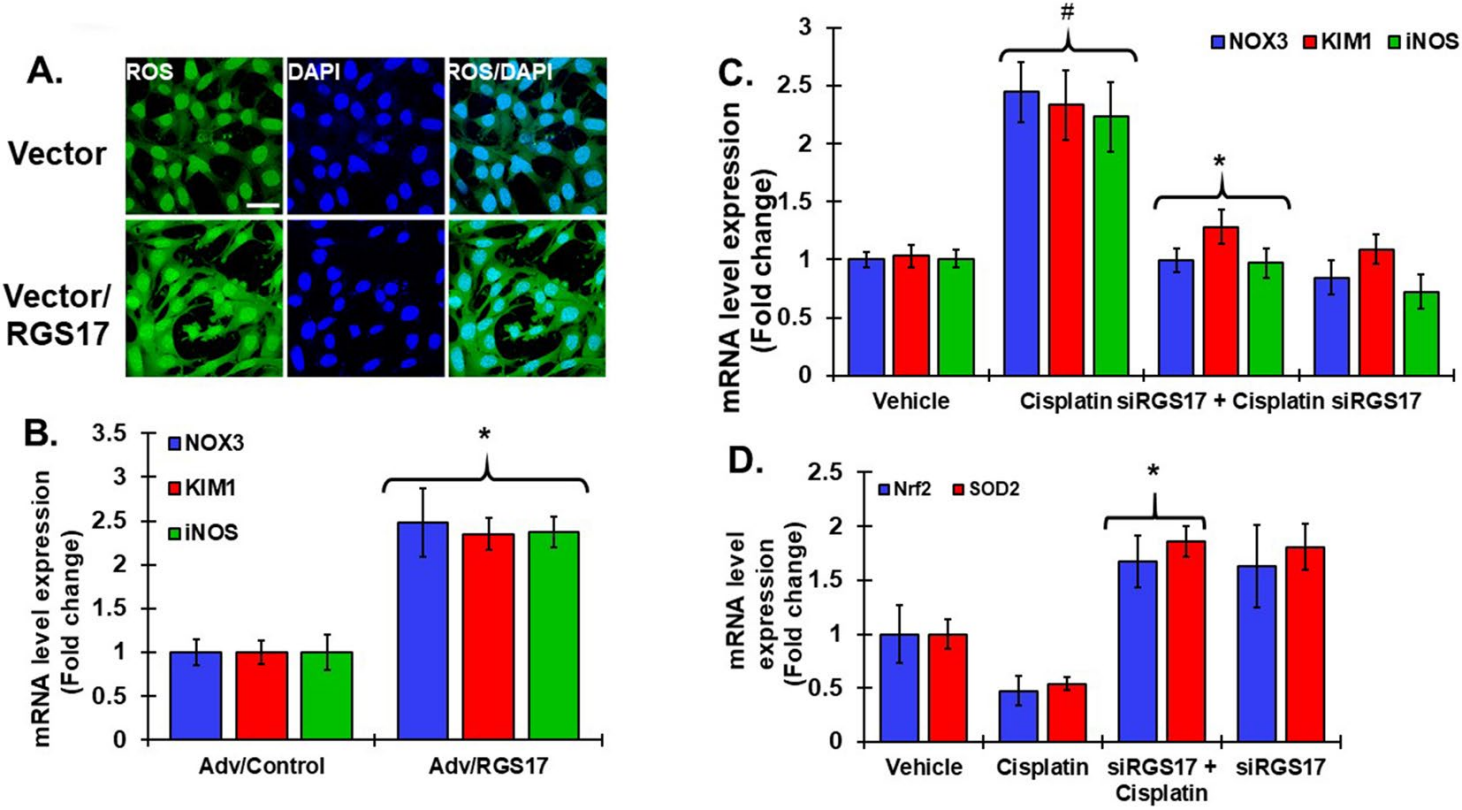
RGS17 regulates oxidative stress in the cochlea. (**A**) UB/OC1 cells were transiently transfected with empty vector or *RGS17* plasmid for 72 h. ROS (green) generation was assessed using the CellROX assay. The cells were incubated with CellROX (5 µM) for 30 min, washed with PBS and fixed in 4% paraformaldehyde, mounted and imaged by confocal microscopy. *RGS17*-transfected cells showed significant increases in ROS. (**B**) Gene expression was assessed via real-time qPCR analysis, conducted in the cochleae collected from treated rats. Expression of oxidative stress genes such as *NOX3*, *iNOS* and *KIM1* significantly increased in cochleae infected with adenoviral vector overexpressing *RGS17* compared to control vector. *p < 0.05 versus control. (**C**) Expression of *NOX3*, *iNOS* and *KIM1* were significantly elevated after cisplatin treatment but was blunted following pretreatment with *siRGS17* administered 2 days prior to cisplatin exposure. (**D**) mRNA levels of antioxidant genes *Nrf2* and *SOD2* were reduced by cisplatin and increased significantly by pretreatments with rats pretreated with *siRGS17*. *siRGS17* added alone elevated the levels of *Nrf2* and *SOD2*. Data indicate fold change in the mRNA levels ± SEM (N ≥ 3).  For (**C**) and (**D**), ^#^p < 0.05 versus vehicle or Adv/control. *p < 0.05 versus cisplatin, two-way ANOVA.

To understand the basis of STAT1 activation and induction by RGS17, we focused on the role of ROS. Cisplatin alters the redox balance in cochlea by increasing the production of ROS which causes cochlear cell damage and apoptosis^6,46^. We hypothesized that induction of *RGS17* potentially increased oxidative stress, tilting redox balance to cell apoptosis. Transiently transfection of UB/OC1 cells with plasmid encoding *RGS17* increased ROS generation, assessed via CellROX assays, as compared to empty vector transfected cells (Fig. 7A). Previous studies have linked cisplatin-induced upregulation of *iNOS*, *NOX3* and *KIM-1* to cause oxidative damage to the cochlea^47–49^. Similarly, we observed increases in these genes in the cochlea following infection with adenoviral vector expressing *RGS17*. In these studies, overexpression of *RGS17* led to increased expression of mRNA level of *NOX3*, *iNOS* and *KIM1* to 2.5 ± 0.4, 2.4 ± 0.2 and 2.4 ± 0.2 fold, respectively (Fig. 7B). In addition, overexpression of *RGS17* in the cochlea significantly increased the levels of inflammatory genes, such as *COX2* and *TNF-α* (see Supplementary Fig. S4A). In additional studies, we administered a scrambled oligonucleotide sequence or *siRGS17* into the cochlea by trans-tympanic injections, waited 2 days and then administered intraperitoneal cisplatin (11 mg/kg). Cisplatin increased the levels of *NOX3*, *iNOS* and *KIM1* by 2.44 ± 0.26, 2.23 ± 0.30 and 2.33 ± 0.29 folds, respectively, while knock down of *RGS17* (by siRNA) reduced these changes to 0.93 ± 0.10, 0.97 ± 0.13 and 1.28 ± 0.14 folds, respectively (Fig. 7C). Moreover, knock down of *RGS17* blocked the reductions in cytoprotective genes such as *Nrf2* and *SOD2* by cisplatin and even promoted the expression of these antioxidant genes to 1.77 ± 0.23 and 1.86 ± 0.14 folds, respectively (Fig. 7D). Knockdown of *RGS17* in the cochlea also significantly reduced cisplatin-induced increase of inflammatory genes, such as *COX2* and *TNF-α* (see Supplementary Fig. S4B). These data suggest that RGS17 serves as a mediator of cisplatin-induced activation of the inflammatory cascade in the cochlea which contributes to hearing loss.

### Reciprocal relation between cannabinoid receptor 2 (CB2) and RGS17

**Figure 8:**
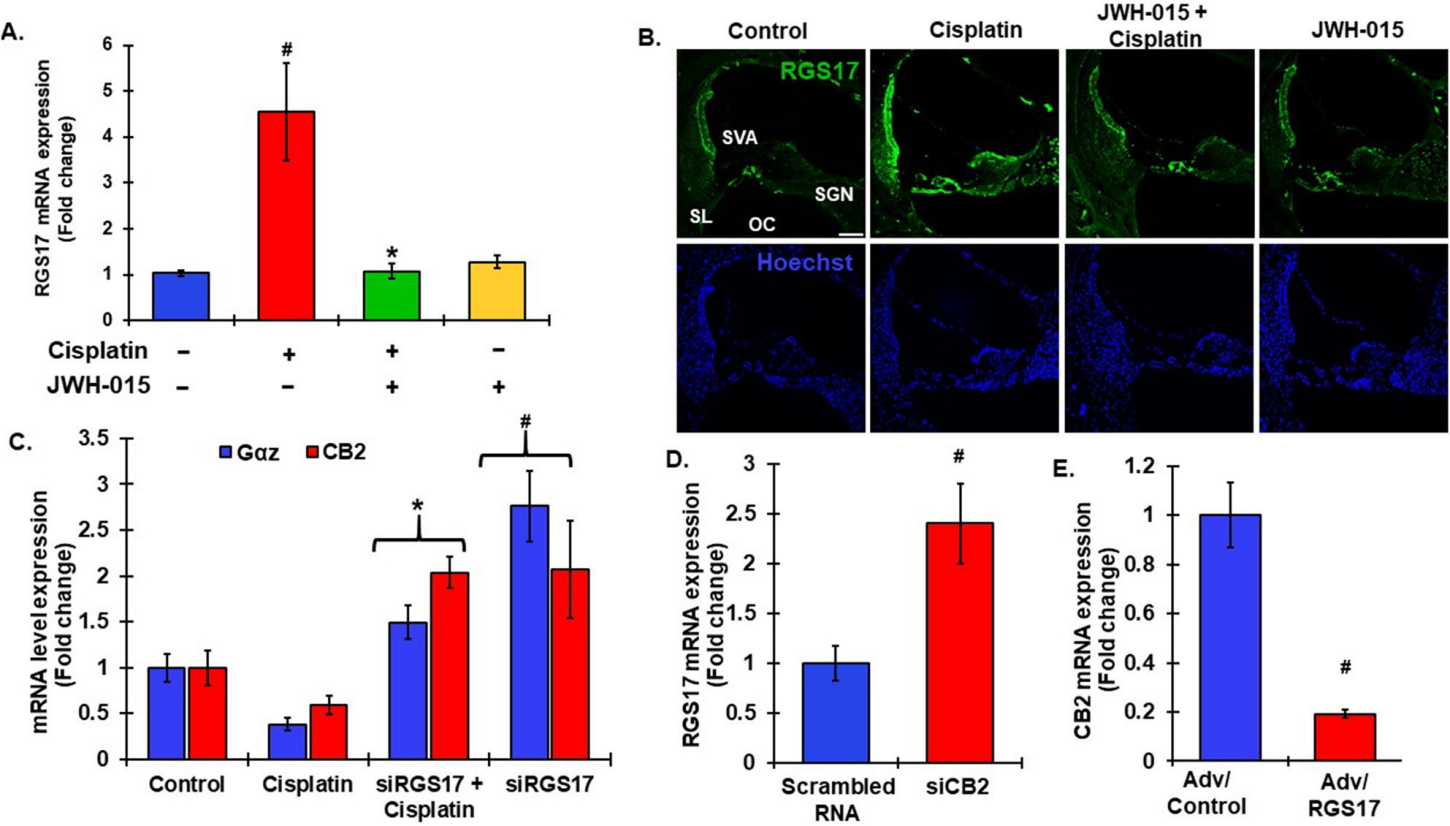
Reciprocal regulation between RGS17 and CB2 signaling. (**A**) Naïve male Wistar rats were treated with CB2 agonist, JWH-015 (0.815 µg/ear) by the trans-tympanic route prior to cisplatin (11 mg/kg, i.p) administration and cochleae were harvested after 3 days. RNA was extracted from the whole cochleae and processed for RT-qPCR. *RGS17* expression was significantly elevated by cisplatin, but was significantly attenuated by pretreatment with JWH-015. #p < 0.05 versus control. *p < 0.05 versus cisplatin, N = 4, two way ANOVA. (**B**) The cochleae collected from same rats were fixed and processed for mid-modiolar sectioning and immunolabelled for RGS17. Cisplatin increased RGS17 immunoreactivity whereas activation of CB2R via JWH-015 reduced this fluorescence. Scale bar 50 μm. (**C**) siRNA against *RGS17*, siRGS17 (0.9 µg/ear), was administered into the rat cochleae 48 h prior to cisplatin exposure. RT-qPCR analysis was performed on the cochleae collected from those treated animals to assess *CB2* and *Gαz* expression. siRGS17 increased cochlear *CB2* and *Gαz* expression whereas cisplatin treatment decreased it. Data indicates mean fold change in expression ± SEM, N ≥ 4, p < 0.01, *statistically significant difference from cisplatin treatments, # indicates statistically significant difference from vehicle control, two-way ANOVA. (**D**) Rats were administered either scrambled RNA (0.9 µg/ear) or siRNA against *CB2* (siCB2R, 0.9 µg/ear) trans-tympanically. Cochleae were collected to perform RT-qPCR analysis. siCB2R treated rats showed significantly increased *RGS17* mRNA expression. #p < 0.05 versus scrambled RNA. (**E**) Conversely, overexpression of *RGS17* in the rat cochlea via administration of adenoviral vector significantly decreased *CB2R* expression. Data represents fold change ± SEM, N ≥ 5t-test p < 0.05, #indicates statistically significant difference from Adv vector control.

A previous study has characterized the role of RGS17 in the central nervous system. They found that cannabinoid receptor 1 interacts with RGS17-HINT complex and this modulatory interaction protected against NMDAR-induced excitotoxicity^50^. A previous study from our lab has indicated the importance of another subtype of cannabinoid receptor, cannabinoid receptor 2 (CB2), against cisplatin ototoxicity^12^. We therefore investigated potential interaction between RGS17 and CB2 in the cochlea. In this regard, we have previously shown that JWH-015, a CB2 agonist, protected against cisplatin-induced hearing loss^12^. In the present study, we show that cisplatin increased the expression of *RGS17* in the cochlea which was abolished by pretreatment of trans-tympanic JWH-015 prior to cisplatin administration (Fig. 8A). qPCR analysis showed a 4.5 ± 1.0-fold increase in *RGS17* with cisplatin, while pretreatment with JWH-015 significantly reduced the fold change to 1.1 ± 0.2. JWH-015 did not produce any change when administered alone but reduced the increase in RGS17 immunoreactivity in cochleae from rats exposed to cisplatin (Fig. 8B). This highlights the role of activated CB2R in suppressing *RGS17* expression, which normally reduces the duration of GPCR-GTP interaction. In addition, in vitro studies showed that transient over expression of *RGS17* in UB/OC-1 cells decreased cell viability to 61.2 ± 3.1%, which was partly reversed by treatment with JWH-015 (cell viability increased to 78.8 ± 3.4) as compared to control vector transfected cells (data not shown). Western blot analysis for G proteins, Gαz and Gαi/o, showed decreases in the levels of Gαz and Gαi/o to 80 ± 10.3% and 86 ± 7.1%, respectively, after cisplatin treatment, whereas JWH-015 treatment increased the levels of Gαz and Gαi/o proteins to 117.3 ± 9.3% and 108.1 ± 6.3% respectively (see Supplementary Fig. S5A–D). These data suggest a close association between the regulation of CB2R, G proteins and RGS17 in regulating cisplatin toxicity in the cochlea.

We next examined the effect of knockdown of *RGS17* on *CB2* expression. Trans-tympanic injection of *siRGS17* before cisplatin treatment significantly elevated *CB2* mRNA level in the cochleae to 2.04 ± 0.20-fold when compared to cisplatin exposed cochleae (0.59 ± 0.10-fold change) (Fig. 8C). Interestingly, knockdown of *RGS17* by itself in the cochlea significantly increased *CB2* expression to 2.07 ± 0.50-fold. In addition, the mRNA level of *GNAZ* (Gαz gene) increased to 2.5 ± 0.2-fold compared to cisplatin treatment (0.4 ± 0.09-fold change), following siRGS17 pretreatment. Both knockdown of *CB2R*^12^ and overexpression of *RGS17* (current data) has been found to exacerbate hearing loss. In this study, we found knockdown of *CB2* with siRNA significantly increased *RGS17* mRNA level by 2.4 ± 0.4-fold (Fig. 8D) and infection of cochleae with adenoviral vector over expressing *RGS17* led to significant reductions in *CB2* expression to 0.2 ± 0.02 fold (Fig. 8E). This shows that the same ototoxic effect observed previously with knockdown of *CB2* and overexpression of *RGS17* may be attributed to induction of *RGS17*. These latter findings suggest a reciprocal regulation between *RGS17* and *CB2*, disruption of which might produce hearing loss.

## Discussion

This study provides evidence implicating RGS17 in cisplatin-induced hearing loss. Evidence to support this conclusion include the demonstration that overexpression of *RGS17* in the cochlea increases cochlear synaptopathy and hearing loss. Cisplatin induced the expression of *RGS17* in the cochlea, which increased oxidative stress, the expression of inflammatory genes and promoted apoptosis of cochlear cells. In contrast, inhibition of RGS17 abrogated cisplatin-mediated oxidative stress and pro-inflammatory genes, reduced cochlear cell apoptosis and preserved synaptic integrity at IHCs. RGS17 and CB2 show reciprocal antagonistic interactions which could influence the otoprotective actions of endocannabinoids and exogenously administered cannabinoids in the cochlea. The transcription factors, STAT1 and STAT3, play a critical role in the reciprocal regulation of *RGS17* and *CB2* in the cochlea by cisplatin. These data show that RGS17 is an important determinant of cisplatin ototoxicity by uncoupling of cochlear GPCRs (such as CB2R) which promotes oxidative stress, inflammation and apoptosis in the cochlea.

Induction of *RGS17* by cisplatin progressed over a 72 h period and was evident in the different regions of the cochlea known to be affected by cisplatin. Knockdown of *RGS17* abrogated cisplatin-induced hearing loss, implicating this protein in hearing loss. Furthermore, overexpression of *RGS17* in the cochlea of naïve rats, using a viral vector, produced hearing loss. The pattern of high frequency hearing loss is somewhat less than that observed with cisplatin and noise exposures in our laboratory^51^. RGS17 mimicked cisplatin by increasing ABR thresholds and producing cochlear synaptopathy. We show that these changes stem from the ability of RGS17 to promote oxidative stress and inflammation, presumably through inactivation of otoprotective CB2R, which is coupled to the Gαi and Gz proteins. We previously observed that CB2R in the cochlea is tonically active and that inhibition of this receptor produced hearing loss^12^. Thus, overexpression of *RGS17* is expected to antagonize the otoprotective actions of CB2R^12^ by rapidly terminating G protein activation mediated by this receptor subtype. Oxidative stress is known to increase lipid peroxidation of membranes, increase inflammation, increase DNA damage and apoptosis, which could contribute to hearing loss. These events could also contribute to synaptopathy, as assessed by reductions in wave I supra-threshold amplitudes^40,41,52^ which we observed in this study. Ghosh et al. observed IHC synaptopathy and ABR threshold shifts following cisplatin treatment which were attenuated by activation of CB2R^12^. We similarly observed that inhibition of RGS17 mimicked the effect of CB2 agonist. These data suggest that boosting the active G-protein signaling either by activating GPCRs (by CB2R agonists) or by delaying the inactivation of GPCR signaling (by inhibiting RGS17), could represent an effective strategy for preserving hearing. Furthermore, these findings highlight the importance of active GPCR signaling process in maintaining normal hearing.

Based on its canonical pathway, RGS17 binds to and promotes the GTPase activity of Gαz, Gαi_1-3,_ Gαo and Gαq^53^. As such, RGS17 can profoundly increase the rate of GTP hydrolysis of these G proteins. For example, RGS17 blocked dopamine (D2) receptor mediated inhibition of cyclic AMP accumulation presumably by acting as a GAP for Gαi/o protein^30^. RGS17 also attenuates μ opioid receptor signaling via Gαz in periaqueductal grey matter^54^ and is able to regulate thyrotropin-releasing hormone receptor by Gα_q/11_. We speculate that a similar action at the level of CB2/Gα could account for the observation that overexpression of *RGS17* by viral vectors or by cisplatin could promote hearing loss. A non-canonical pathway for RGS17 described in the brain shows its interaction with HINT1 complex which couples mu opioid receptors to protein kinase C-γ and facilitates activation of the ERK-MAP kinase pathway through NO-dependent release Zn^2+^ ions^55^. Such a mechanism is linked to opioid tolerance^55^ and could similarly contribute to desensitization of CB2R to endocannabinoids in the cochlea.

We show that overexpression of *RGS17* in the cochlea is associated with increased oxidative stress and inflammatory proteins, NOX3, KIM1 and iNOS, an action which is characteristic of inhibition of CB2R in the cochlea^12^. This likely represents uncoupling of CB2R from its cytoprotective effectors which is blunted by knockdown of *RGS17*. We show that one of the downstream targets of RGS17 is STAT1 which contributes to cochlear inflammation^56^. Studies have established STAT1 as a common mediator for inflammation, apoptosis and hearing loss^11,56^. In addition, the cellular STAT1/STAT3 balance is crucial to maintain apoptosis-survival homeostasis in the cochlea^11^. Similar to cisplatin, we show that overexpression of *RGS17* increased Ser^727^ STAT1 phosphorylation and decreased Tyr^701^ STAT3 phosphorylation, elevating the STAT1/STAT3 ratio which promotes cell apoptosis. Interestingly, the promoter of *RGS17* gene has a STAT1 binding site^45^, suggesting that this mechanism could confer induction of *RGS17*. In preliminary studies, we show that the STAT1 inhibitor, EGCG, significantly decreased *RGS17* expression (unpublished data). Thus, there likely exists a pathway from ROS to STAT1 and the induction of *RGS17*, which ultimately generates more ROS and RGS17.

Glutamate excitotoxicity is suggested to be a key mediator in noise-induced cochlear synaptopathy^57,58^. Glutamate agonists produced swollen cochlear nerve terminals and neuronal damage similar to noise exposure, whereas glutamate antagonist protected cochlear auditory neurons against excitotoxicity^57,59,60^. Thus, the cochlear synaptopathy observed in cisplatin-treated rats could also be due to the excessive glutamate release or NMDAR hyperactivity at IHC. In addition, activation of presynaptic CB2R in the CNS inhibits glutamate release at subthalamo-nigral synapses via the Gαi subunit^61^. It is possible that presynaptic inhibition of glutamate release at IHCs by CB2R activation prevents cisplatin-induced synapse loss, as observed by Ghosh et al.^12^. Chronic activation of NMDAR upregulates TNF-α, IL-1β, iNOS and arachidonic acid levels^62,63^ which can, in turn, block reuptake of glutamate at synapses, thereby compounding glutamate excitotoxicity at synapses^64^. Thus, a pathway exists between inflammation and excitotoxicity which can promote neuronal damage and synapse loss^64^. In this study, we observed increased inflammatory markers in the cochlea following overexpression of *RGS17*, whereas knockdown of *RGS17* reduced the inflammation and also promoted synaptic preservation (comparable to activation of CB2R). How RGS17 regulates the inflammatory process and the identity of immune cells involved in synaptic remodeling was not addressed in this study. However, since reductions in tonic CB2R activity using an antagonist produce inflammation, oxidative and synaptic loss^12^, one can reasonably assume that a similar sequence of events would accompany overexpression of *RGS17*. A likely scenario is that RGS17 uncouples CB2R/G protein and reduces its function and thereby acts indirectly like a CB2R antagonist. Taken together, we believe that these results demonstrate a constitutive role of CB2R/RGS17/G protein signaling in preserving IHC synapses.

Two interesting observations from our study is that the expression of *RGS17* is under control of cisplatin and that RGS17 negatively influences *CB2R* expression. These data suggest a reciprocal interaction between RGS17 and CB2R such that both proteins could likely serve as therapeutic drug targets for treating cisplatin-induced and possibly other forms of hearing loss. In this regard, our data show that siRGS17 inhibited cisplatin-induced hearing loss, synapse loss and apoptosis of cochlear cells while trans-tympanic administration of CB2R agonist (JWH-015) protected against cisplatin-induced hearing loss^12^. In contrast, overexpression of *RGS17* in the cochlea or blockade of CB2R produced hearing loss. An unanticipated finding is that trans-tympanic administration of siRNA against *RGS17*, by itself, stimulates the expression of *CB2R*, *Gzα*, *Nrf2* and *SOD2*. These finding are intriguing in that they suggest that these genes are all under tonic regulation by RGS17. While the mechanism(s) underlying induction of these genes is unclear, increases in these proteins could enhance the endogenous otoprotective actions of endocannabinoids in the cochlea.

**Figure 9:**
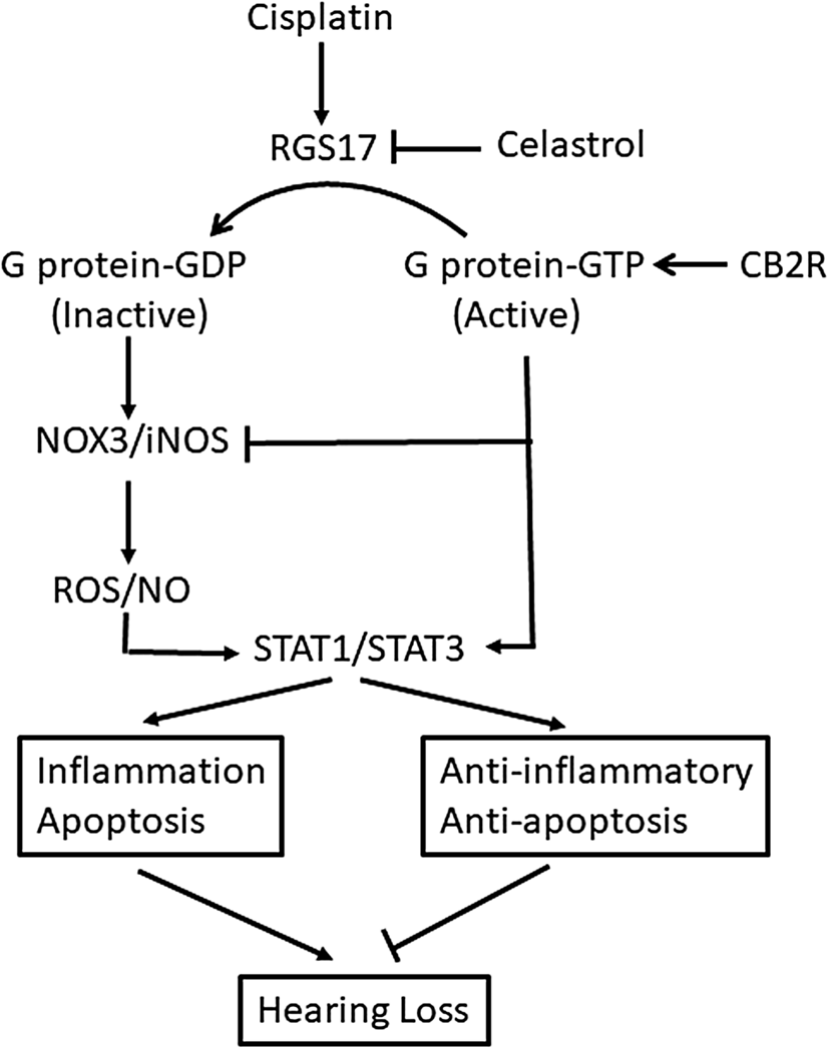
Proposed mechanism underlying the involvement of RGS17 in cisplatin ototoxicity. In the cochlea, cisplatin-induced increase in RGS17 negatively modulates otoprotective GPCR/Gα signaling by facilitating the inactive Gα-GDP state. This, we believe relieves a tonic suppression of ROS and reactive nitrogen species (RNS) generation via NOX3 and iNOS, respectively, increases cellular STAT1/STAT3 ratios and increases pro-inflammatory and apoptotic pathways. We believe these changes promote hearing loss. Inhibition of RGS17 (by celastrol, knockdown of *RGS17*) or activation of CB2 relieves the oxidative stress, reduced the STAT1/STAT3 ratios and inhibits cochlear inflammation and apoptosis.

In summary, our study identified a new gene, *RGS17*, which is implicated in cisplatin-induced hearing loss (proposed mechanism see Fig. 9). We showed that *RGS17* is induced by cisplatin which could render GPCRs (such as CB2R) less responsive to endogenous and exogenous agonists. Furthermore, knockdown or inhibition of this protein protected against cisplatin-induced hearing loss. Importantly, RGS17 antagonizes the otoprotective action of CB2R and suppresses its expression. Thus, RGS17 could represent a novel target for treating cisplatin-induced hearing loss and potentially other toxicities associated with this drug.

## Materials and methods

### Drugs and antibodies

Cisplatin and celastrol (3-hydroxy-9β, 13α-dimethyl-2-oxo-24, 25, 26-trinoroleana-1(10), 3, 5, 7-tetraen-29-oic acid) were purchased from Sigma Aldrich (St. Louis, MO). CB2 agonist, JWH-015^12^ was purchased from Tocris Biosciences (R&D System, MN). Primary antibodies used are listed as follows: RGS17 (#12549-1-AP) from Proteintech group, IL. Two different myosin VIIa antibodies of two different species were used: Mouse IgG1 anti-myosin-VIIa antibody from Developmental Studies Hybridoma Bank (71-1-s), while rabbit anti-myosin-VIIa from Proteus Biosciences (71-6790) was purchased. Anti-CtBP2 (#612044) and anti-GluR2 (#MAB397) were obtained from BD Biosciences Millipore respectively. Secondary antibodies: Alexa Fluor 647 goat anti-rabbit, Alexa Fluor 488 goat anti-rabbit were purchased from Life Technologies whereas DyLight 488 and TRITC conjugated secondary antibodies were purchased from Jackson lmmuno Laboratories (West Grove, PA) were used for immunocytochemistry whereas donkey anti-rabbit IRDye 680RD, goat anti-mouse IRDye 800RD (no. 926–32,214) from LI-COR Biosciences (Lincoln, NE, USA) were used for western blots. *RGS17* siRNA was obtained from Invitrogen - Thermo Fisher Scientific, USA.

### Animals

This study was performed in compliance with the ARRIVE guidelines. Adult male Wistar rats (weighing ~ 200 g) were purchased from Envigo, Indianapolis (USA) and housed at the animal facility at the Southern Illinois University School of Medicine, IL. Rats were housed in a temperature-controlled room with normal light/dark cycles (12 h) with easy access to food and water ad libitum. Auditory brainstem recordings (ABRs) were performed in rats anesthetized using a 3:1 mixture of ketamine (90 mg/kg) and xylazine (17 mg/kg). These studies were generally performed between 9 AM and 12 Noon and animals were returned to the vivarium after they had fully recovered from anesthesia. Pre-ABRs were performed prior to any trans-tympanic treatments on anesthetized rats. Rats were administered cisplatin (11 mg/kg) by intraperitoneal injections and final ABR measurements were performed 72 h later. Rats were then immediately sacrificed by decapitation under anesthesia. And cochleae dissected out for RNA extraction in RNAlater (Thermo Fisher Scientific) or fixed using cochlear perfusion of 4% paraformaldehyde in PBS. All animal studies were approved by the Southern Illinois University School of Medicine Laboratory Animal Care and Use Committee (LACUC) and monitored by the ethics and regulation protocol of SIU School of Medicine LACUC.

### Cell culture

Immortalized organ of Corti cell line, UB/OC-1, derived from transgenic mouse (Immortomouse), was provided by Dr. Mathew Holley (Institute of Molecular Physiology, Western Bank, and Sheffield, UK. The cells were cultured in RPMI 1640 (modified with l-glutamine and phenol red) supplemented with 10% FetalClone II serum (HyClone), 5% penicillin–streptomycin and 1% Normocin (Invitrogen). Cells were incubated at 33 °C with 10% CO_2_. Confluent monolayer cells were passaged thrice a week and were used for experiment within passage 3 to 8.

### Trans-tympanic administration of siRNA and adenoviral vector

Trans-tympanic injections were performed in the anterior inferior region of rat’s tympanic membrane using a 28-30G needle with the aid of a Zeiss operating microscope. Following injections, rats were kept undisturbed with the injected ear facing upward for at least twenty min. Injections included 0.9 μg of siRNA in 50 μl of nuclease free water per cochlea or 4 × 10^8^ p.f.u/cochlea of adenoviral vector. This protocol has been previously described by our lab^65^.

### Auditory brain response (ABR)

ABRs were determined as described previously^66^. Male Wistar rats were anesthetized (as above) and electrode placement locations include the vertex of the skull (active), hind flank muscle (ground) and pinna (two reference electrodes under each pinna). Sound stimului were tone bursts at 8, 16 and 32 kHz with a 5 ms plateau and a 1 ms rise/fall time presented at a rate of 5/s. The auditory stimulus intensity was initiated at 10 dB sound pressure level (SPL) and reaching to a maximum intensity at 90 dB SPL with 10 dB increments. The auditory threshold was determined as a minimum sound intensity evoking visually detectable waves II and III, with a minimum amplitude of 0.5 μV. Threshold shift are calculated based as the difference between pre- and post-treatment thresholds obtained at each frequency.

### Detection of ROS

ROS at cellular level was measured in UB/OC-1 cells using CellROX assay (Invitrogen), as described previously^11^. Cells were plated in 12 well plate with coverslips and pre-treated with vehicle or drugs or transfected with different agents. CellROX reagent was added at a final concentration of 5 μM and coverslips were incubated for 30 min at 33 °C. Coverslips were washed with PBS and fixed using 4% paraformaldehyde for 15 min and mounted on glass slides using VECTASHIELD mounting medium with DAPI (Vector Laboratories). ROS fluorescence was captured with a Leica Confocal microscope at 488 nm.

### Apoptosis detection by flow cytometric analysis of cells

Apoptosis in cell cultures was detected using an FITC Annexin V Apoptosis detection kit (BD Pharmingen, BD Biosciences) as described previously^11^. Briefly, following treatments, UB/OC1 cells were washed with ice cold 1× PBS, trypsinized and then resuspended in 1× binding buffer provided in the kit. Cells were transferred into a 5 ml falcon tubes to which were added 5 µl of both FITC Annexin V and propidium iodide (PI) and gently vortexed. The cells were then incubated for 15 min at room temperature in the dark. Binding buffer was mixed into each tube prior to flow cytometry (BD FACSCalibur). Controls include unstained cells, cells stained with either FITC Annexin V (no PI) or PI (no FITC Annexin V). Data are analyzed using the CellQuest software provided with the FACSCalibur.

### MTS assay for cell viability

UB/OC-1 cell proliferation was measured using CellTiter 96 AQueous One Solution Cell Proliferation Assay kit (Promega, Madison, WI), as described previously^11^. UB/OC-1 cells were plated in 96-well plate (3000 cells per well). Following treatments, 20 µl of Cell Titer Aqueous One Solution reagent was added to each well of the 96-well plate containing 100 µl of culture media. Assay plates were incubated at 33 °C for 1–2 h in humidified, 5% CO2 atmosphere. Absorbance was recorded at 490 nm using Fluoroskan Ascent FL Microplate Fluorometer Plate reader (Thermo Scientific). Percentage of cell viability is calculated by normalizing to the control group.

### Western blot

Western blotting in cell cultures was performed as described previously^11^. UB/OC1 cells were lysed using RIPA buffer (50 mM Tris, 150 mM NaCl, 0.1% SDS, 0.5% sodium deoxycholate, 1% NP-40, pH 8.0) containing protease inhibitor (1:100, Sigma-Aldrich) and phosphatase inhibitors (Cocktail A and B, 1:100, Sigma-Aldrich), centrifuged at 4 °C 14,000 rpm for 10 min and supernatant was collected. Protein concentrations were determined using Bradford reagent^67^ (MilliporeSigma). 60–80 μg of the collected whole cell lysate was then mixed with 5× Laemmli buffer (0.5 mM Tris–HCl pH6.8, glycerol, SDS, 0.25% bromophenol blue and beta-merceptoethanol) and heated at 90 °C for 5 min, loaded onto a 10–14% SDS PAGE gel. Proteins were then transferred to nitrocellulose membranes which were incubated with blocking buffer (10 mM PBS, 10 mM EDTA, 20% TritonX-100 and 5% bovine serum albumin) for 1 h and followed by incubation with primary antibody overnight and at 4 °C. The membranes were washed with 1× TBST (20 mM Tris pH 7.5, 150 mM NaCl, 0.1% Tween) and exposed to secondary antibodies for 1 h at room temperature and then imaged using LICOR Odyssey Image X. Proteins bands were analyzed using the Odyssey software normalized to actin (or total STAT1 or total STAT3 where relevant).

### RNA isolation

This procedure was performed essentially as described previously^11^. Snap frozen cochleae were crushed in 1 ml of TRIzol reagent (Sigma-Aldrich) using a homogenizer and incubated in the reagent at room temperature for 15 min. RNA isolation from cells, TRIzol was added to each well of the cell cultured dish and the solution transferred to a microfuge tube and vigorously vortexed for 30 s. This was followed by a chloroform extraction step. Sample tubes were then centrifuged at 12,000×*g* for 15 min at 4 °C. The aqueous phase (clear solution) containing RNA was collected carefully and transferred to DNase RNase free clear microfuge tube and 100% ice-cold isopropanol (500 µl) was added. Tubes were incubated overnight at − 80 °C for precipitation of RNA, centrifuged at 12,000×*g* for 20 min at 4 °C and formed RNA pellet was washed once with 100% molecular grade ice-cold ethanol and twice with 70% ethanol followed by 15 min centrifugation each times. The ethanol was removed, the RNA pellet resuspended in warm nuclease-free water and its concentration determined by a NanoDrop ND-1000 spectrophotometer. The pure RNA samples (260/230 wavelengths ranging from 1.8 to 2) were used for PCR.

### Real time Q-PCR

These assays were performed as described previously^11^. Total RNA (500 ng) was used to prepare gene specific cDNA by using iScript Select cDNA Synthesis Kit (Bio-Rad). cDNA was synthesized using thermal cycler following reaction conditions at 42 °C for 60 min and 85 °C for 5 min. This cDNA reaction mix was used for q RT-PCR which was performed by using StepOnePlus Real-Time PCR System (Thermo Fisher Scientific). The cycling conditions used were 95 °C for 3 min followed by 45 cycles at 95 °C for 15 s, 58–64 °C for 30 s (depending on primer) and 95 °C for 3 s. Upon completion of amplification protocol, the melting curves were analyzed by cooling the reaction to 60 °C and slowly reheating to 95 °C. The mRNA expression were normalized to the levels of housekeeping gene, GAPDH. Negative controls were set up for both the target and housekeeping gene and no template cDNA was added to this mixture. The relative change in mRNA levels of a specific gene between the vehicle control and treated group was quantified by using the formula: 2^(Ct Target gene1-Ct GAPDH)-(Ct Target) gene)2-GAPDH2)^^68^. The nucleotide sequences of rodent primer sets were based on homologous sequences of rat and mouse cDNA sequence (see Supplementary Table S1).

### Cochlear whole-mount preparation for OHC and ribbon synapse counts

The studies were performed as described previously^12^. Rat cochleae was decalcified using 120 mM ethylenediaminetetraacetic acid (EDTA) for about 3 weeks in a rotating device. The organ of Corti was carefully microdissected from each decalcified cochlea and divided into the apical, middle and basal turns. Sections were immunolabeled with antibodies against myosin VIIa, CtBP2, and GluR2 for staining of hair cells, presynaptic ribbon and post-synaptic glutamate receptors, respectively. The number of OHCs per every 100 µm of the sections was counted manually and reported as an average number of OHC/100 µm. Zeiss LSM800 (× 63 magnification) was used to capture 10 IHCs per image. Three random sections from one cochlear whole mount sample were chosen to evaluate presence of functional synapse and aired synaptic ribbons (CtBP2 + GluR2 immunolabeling overlapping each other) were counted manually from each image.

### Immunohistochemistry

Mid-modiolar section (10 μm thickness) were prepared from decalcified cochleae, permeabilized with 100% ice-cold ethanol and blocked in 1% Triton X-100, 1% BSA and 10% goat or donkey serum for 1 h at room temperature. The specimen were then incubated in primary antibody overnight at 4 °C. The following antibodies were used: anti-myosin7A (mouse IgG1; 1:50), anti-myosin7A (rabbit; 1:200), anti-RGS17 (rabbit; 1:100), anti-Tuj1 (mouse IgG2a; 1:500), anti-GluR2 (rabbit) and anti-CtBP2 (1:200). Samples were then washed 3 times with 1X PBS for 10 min and incubated in secondary antibodies on a rotator for 3 h in dark at room temperature and then washed three times for 10 min with 1× PBS. Sections were then stained with Hoechst (1:2000) for visualizing nuclei staining, washed and mounted on slide using ProLong Diamond Antifade Mountant (Invitrogen). The slides were imaged by Leica SP5 II or Zeiss LSM800 scanning confocal microscope.

### TUNEL staining of cochlear sections

Apoptosis will be detected, as previously described^11^, by TUNEL assay using ApopTag Red in Situ Apoptosis Detection Kit (Millipore sigma, USA) and according to the manufacturer’s instructions. Briefly, cochlea cryosections were permeabilized using ethanol:acetic acid and then incubated with TdT enzyme for 1 h at 37 °C. Sections were then washed in stop buffer for about 10 min at room temperature and incubated with an anti-digoxigenin conjugate (rhodamine) for 30 min. Slides were counterstained with Alexa Fluor 488 phalloidin conjugate antibody, followed by Hoechst staining for visualization of cell nuclei. Images were captured using a Leica Laser Scanning Confocal Microscope. TUNEL positive cells in organ of Corti, stria vascularis, spiral ligament and spiral ganglion cells are determined by counting of red fluorescent cells (TUNEL positive) from three different cochlear samples.

### Statistics

Data are presented as mean ± standard error of mean (SEM). Statistical significance of differences among groups were tested using t-test, one-way or two-way analysis of variance (ANOVA) depending upon experiments, followed by TUKEY’s post hoc analysis, using GraphPad Prism version 6.07 for Windows. p value < 0.05 was considered significant.

## Supplementary Information

Supplementary Information.
