# Supplementary material for: Regulator of G protein signaling 17 represents a novel target for treating cisplatin induced hearing loss
Source: Sci Rep. 2021 Apr 14;11:8116. doi: 10.1038/s41598-021-87387-5 (PMC8046767; doi:10.1038/s41598-021-87387-5)
Supplement: Supplementary file 1 — Supplementary Information. [file 41598_2021_87387_MOESM1_ESM.pdf]

Supplementary Figures and Table:

Regulator of G Protein Signaling 17 Represents a Novel Target for Treating Cisplatin Induced Hearing Loss

Asmita Dhukhwa[1], Raheem F H Al Aameri[1], Sandeep Sheth[3], Debashree Mukherjea[2], Leonard Rybak[2] and Vickram Ramkumar[1*]

[1] Department of Pharmacology, Southern Illinois University School of Medicine, Springfield, IL 62702

[2] Department of Otolaryngology, Southern Illinois University School of Medicine, Springfield, IL 62702,

[3] Department of Pharmaceutical Sciences, Larkin University College of Pharmacy, Miami, FL 33169,

[*]Corresponding author: vramkumar@siumed.edu

Supplementary Figures:

Supplementary Figure 1:

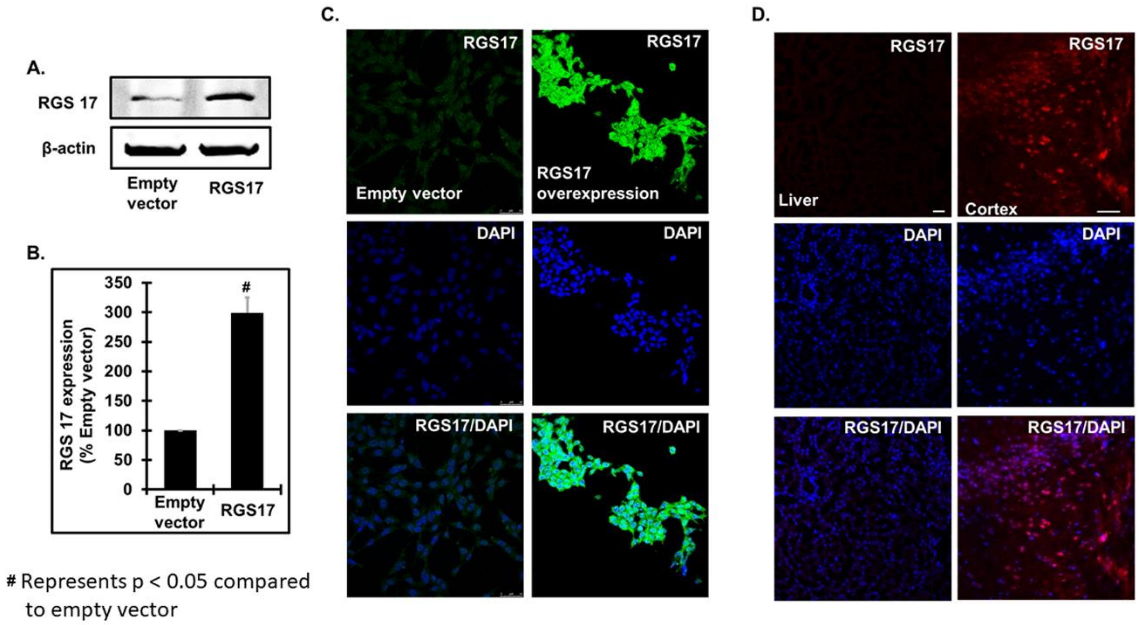

Supplementary Figure 1. Verification and validation of RGS17 antibody. **A.** UB\OC-1 cells were transfected with either empty vector (control) or RGS17 overexpression vector for 48-72 hours, cell lysates were prepared and immunoblotted using anti-RGS17 antibody. RGS17-transfected cells show more immunoblot reactivity when compared to the control. **B.** Graphical representation of western blot analysis shows significant increase in RGS17 expression in RGS17-transfected cells. **C.** Both RGS17-transfected cells and vector control cells were stained using RGS17 antibody. Immunofluorescence activity of RGS17-overexpressed cells is higher in intensity than control cells. **D.** Liver and cerebellum was harvested from C57/BL6 mice and fixed it using 4% paraformaldehyde. Tissues were cryo-sectioned and stained with anti-RGS17 antibody. Cortex shows higher intensity fluorescence of RGS17 compared to liver. (Scale 50 μm).

Supplementary Figure 2:

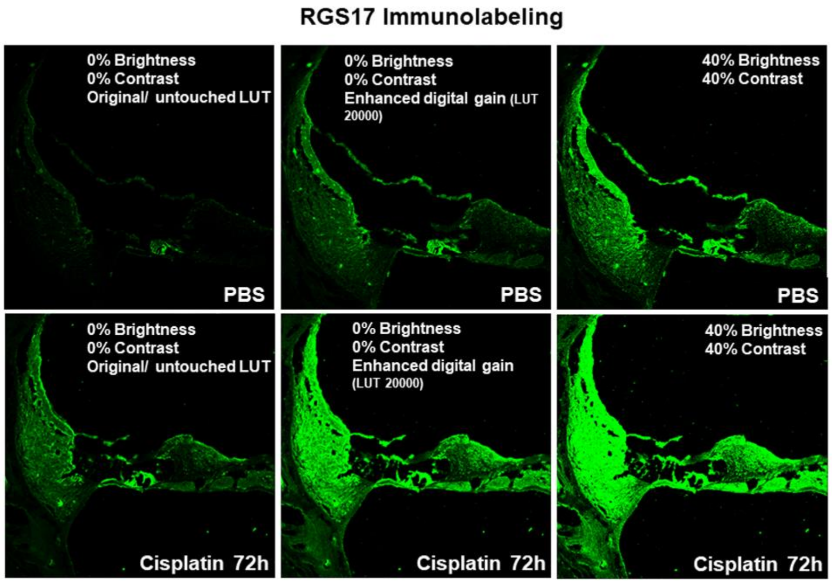

Supplementary Figure 2. Immunolabeling of mid-modiolar section of the rat cochlea showing modification of original untouched LUT images versus enhanced digital gain and enhanced brightness and contrast.

Supplementary Figure 3:

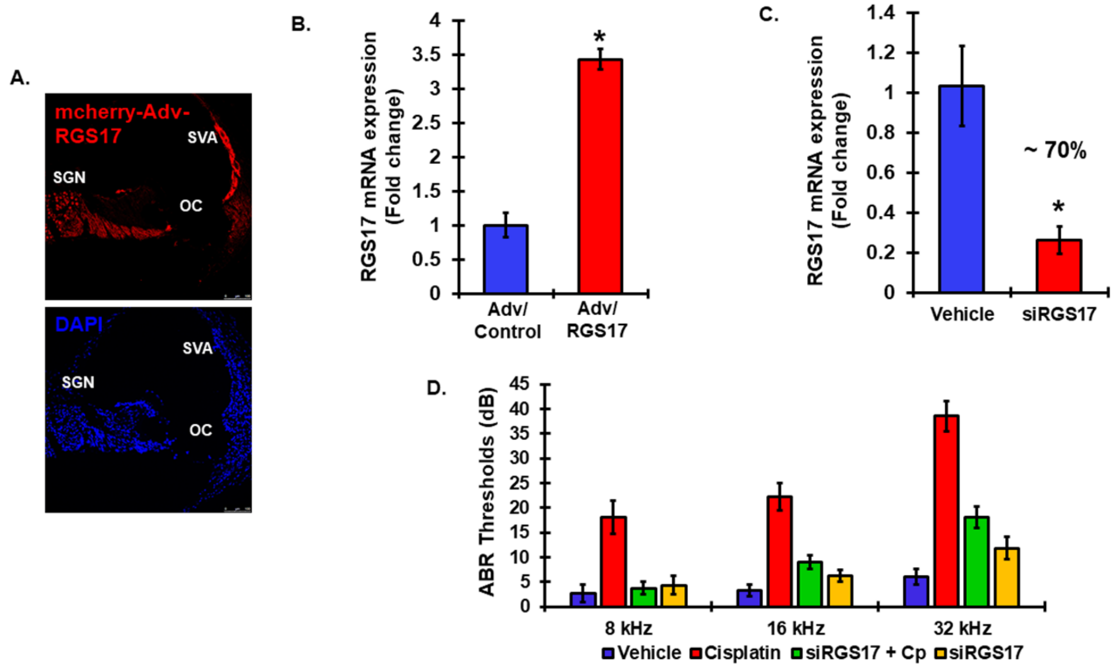

<u>**Supplementary Figure 3.**</u> <u>Quantification of overexpression and knockdown of *RGS17* and ABR thresholds of *RGS17* knockdown animals.</u> Cochleae from treated male Wistar rats were processed for RNA isolation. Four group of animals: adenoviral vector control, *RGS17* overexpressing adenoviral vector (Adv), vehicle and siRGS17 treated groups. **A.** *RGS17* overexpressing adenoviral vector was tagged with mcherry. Rats were administered adenovirus overexpressing *RGS17* via trans-tympanic route. The cochleae were collected, fixed and processed for midmodiolar sectioning. They were stained with anti-mcherry (red) antibody to detect mcherry fluorescence which indicated *RGS17* expression after infection. **B.** Trans-tympanic administration of Adv overexpressing *RGS17* significantly increases the expression of *RGS17* mRNA level in the cochlea by more than 300% whereas **C.** siRGS17 administration significantly decreased *RGS17* mRNA level which was about 70% knockdown. Data represents mean fold change ± SEM, N=6, * denotes statistically significant difference from controls, $p<0.05$. **D.** Post ABR results showed significant elevations in ABR threshold shifts with cisplatin at 8, 16 and 32 kHz. siRGS17 pretreatment significantly attenuated cisplatin-induced elevation in ABR threshold shifts. Data indicate mean ± SEM. Asterisks, $^{\#}p<0.01$ vs. vehicle, $^{*}p<0.01$ vs. cisplatin, N>12, two-way ANOVA. TT: trans-tympanic; i.p: Intraperitoneal.

Supplementary Figure 4:

Supplementary figure 4

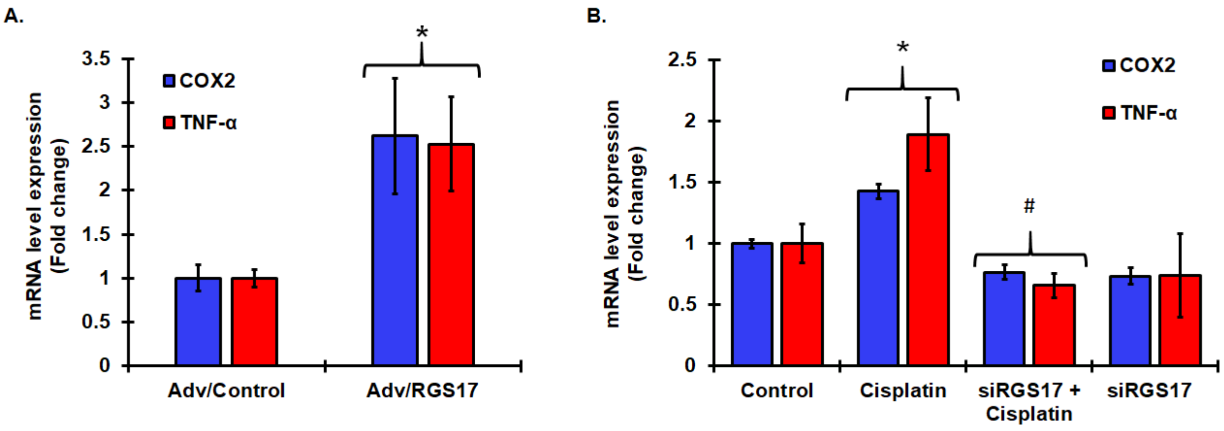

<u>Supplementary Figure 4.</u> <u>Overexpression of *RGS17* in the cochlea elevates inflammatory mediators whereas knockdown of *RGS17* attenuates it.</u> RNA collected from the animals described in fig. was used to conduct RT-qPCR for inflammatory genes. **A.** Cochleae overexpressing *RGS17* significantly increased expression of *COX2* and *TNF-α* as compared to control vector group. **B.** Cisplatin treated rats showed significant increase in *COX2* and *TNF-α* expression in the cochleae as compared to vehicle treated rats whereas siRGS17 pretreated animals significantly decreased mRNA expression of these inflammatory genes as compared to cisplatin treated cochleae. Data indicate fold change in the mRNA levels ± SEM, N≥4, statistical significance #$p<0.05$ vs vehicle and *$p<0.05$ vs cisplatin group, two-way ANOVA.

Supplementary Figure 5:

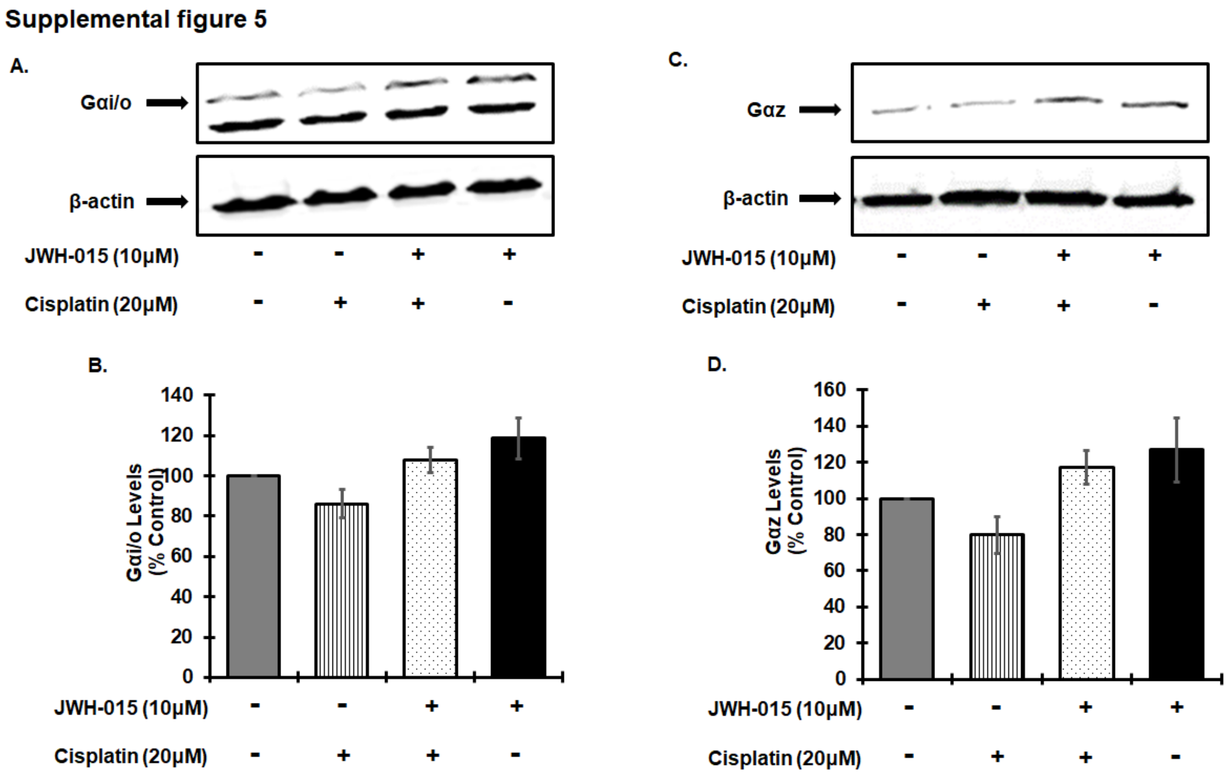

<u>Supplementary Figure 5.</u> <u>Activation of CB2R increases the expression of *Gα* proteins *Gαi/o* and *Gαz* expression *in vitro*.</u> UB/OC1 cells were pre-treated with either vehicle (PBS) or JWH-015 (10μM) for 30 min followed by cisplatin (20μM) treatment for 24 hours and cell lysates were prepared. The levels of Gαi/o and Gαz was analyzed using western blot analysis. Normalization of these Gα protein was performed against beta-actin. **A and B.** Western blot analysis showed cisplatin treatment decreased Gαi/o, but JWH-015 pretreatment induced the levels of Gαi/o. **C and D.** Similarly, western blot analysis showed cisplatin decreased Gαz, whereas pretreatment with JWH-015 increased the levels of Gαz. Data represents mean ± SEM, N=3.

Supplementary Table 1: PCR Primers Sequences Used

Rodent-TNF-α	Forward	5′-CAGACCCTCACACTCAGATCA-3′
Rodent-TNF-α	Reverse	5′-TGAAGAGAACCTGGGAGTAGA-3′
Rodent-COX-2	Forward	5′-TGATCGAAGACTACGTGCAAC-3′
Rodent-COX-2	Reverse	5′-GTACTCCTGGTCTTCAATGTT-3′
Rodent-NOX3	Forward	5′-ACC AGG CAA TTC ACA TAG CT -3′
Rodent-NOX3	Reverse	5′-CCA CAG AAG AAC ACG CCA A -3′
Rodent-KIM1	Forward	5′- ATGTGCTTGTCACCACCAG -3′
Rodent-KIM1	Reverse	5′-TTCAAGTCTTCATTTCAGGCC -3′
Rodent-STAT1	Forward	5′-CAT GGA AAT CAG ACA GTA CCT -3′
Rodent-STAT1	Reverse	5′-TCT GTA CGG GAT CTT CTT GGA -3′
Rodent-STAT3	Forward	5′-CAG CCA AAC TCC CAG ATC AT -3′
Rodent-STAT3	Reverse	5′-ACC CAG ATT GCC CAA AGA TAG -3′
Rodent-RGS17	Forward	5′-GAA GTC TTG TCC TGG TCT CA -3′
Rodent-RGS17	Reverse	5′-TTG ATC ACC TCT CTA ACT CG -3′
Rodent-iNOS	Forward	5′-CAT TCT ACT ACT ACC AGA TC-3′
Rodent-iNOS	Reverse	5′- ATG TGC TTG TAA CCA CCA G-3′
Rodent-SOD	Forward	5′-GCC TCA GCA ATG TTG TGT C -3′
Rodent-SOD	Reverse	5′-TCA GAT TGT TCA CGT AGG TC -3′
Rodent-NrF2	Forward	5′-CTC TCT GGA GAC GGC CAT GAC T -3′
Rodent-NrF2	Reverse	5′-CTG GGC TGG GGA CAG TGG TAG T -3′
Rodent-GNAZ	Forward	5′-GAC TCC ATC TGC AAC AAC AAC -3′
Rodent-GNAZ	Reverse	5′-TTC CTC GTA CGT GTT CTG AC-3′
Rodent-GAPDH	Forward	5′-ATG GTG AAG GTC GGT GTG AAC-3′
Rodent-GAPDH	Reverse	5’-TGT AGT TGA GGT CAA TGA AGG -3′

Supplementary Information:

Regulator of G Protein Signaling 17 Represents a Novel Target for Treating Cisplatin Induced Hearing Loss

Asmita Dhukhwa[1], Raheem F H Al Aameri[1], Sandeep Sheth[3], Debashree Mukherjea[2], Leonard Rybak[2] and Vickram Ramkumar[1*]

[1] Department of Pharmacology, Southern Illinois University School of Medicine, Springfield, IL 62702

[2] Department of Otolaryngology, Southern Illinois University School of Medicine, Springfield, IL 62702,

[3] Department of Pharmaceutical Sciences, Larkin University College of Pharmacy, Miami, FL 33169,

[*]Corresponding author: vramkumar@siumed.edu

Regular and Supplementary Figures – Full Gel Images

Figure 2A:

- Fluorescence western blotting technique was used to detect protein band
- Images were taken using Odyssey Imaging system by LI-COR biosciences

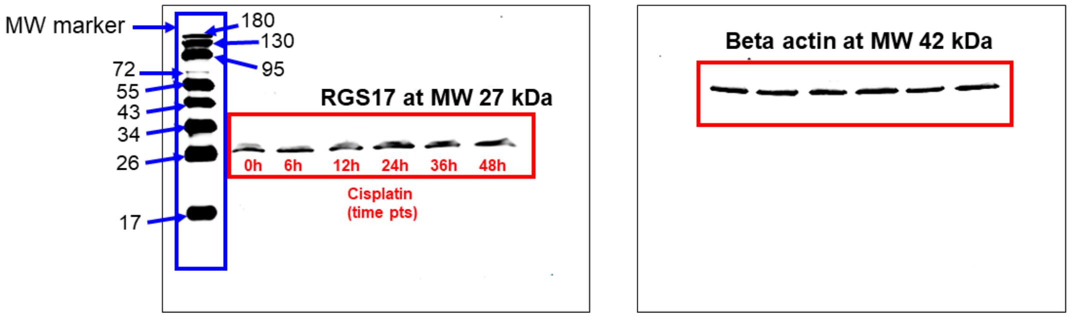

Figure 2B:

- RGS17 protein was detected via Chemiluminescence western blotting technique
- Beta actin protein was detected via Fluorescence western blotting technique using IR dye 800 channel (green color)

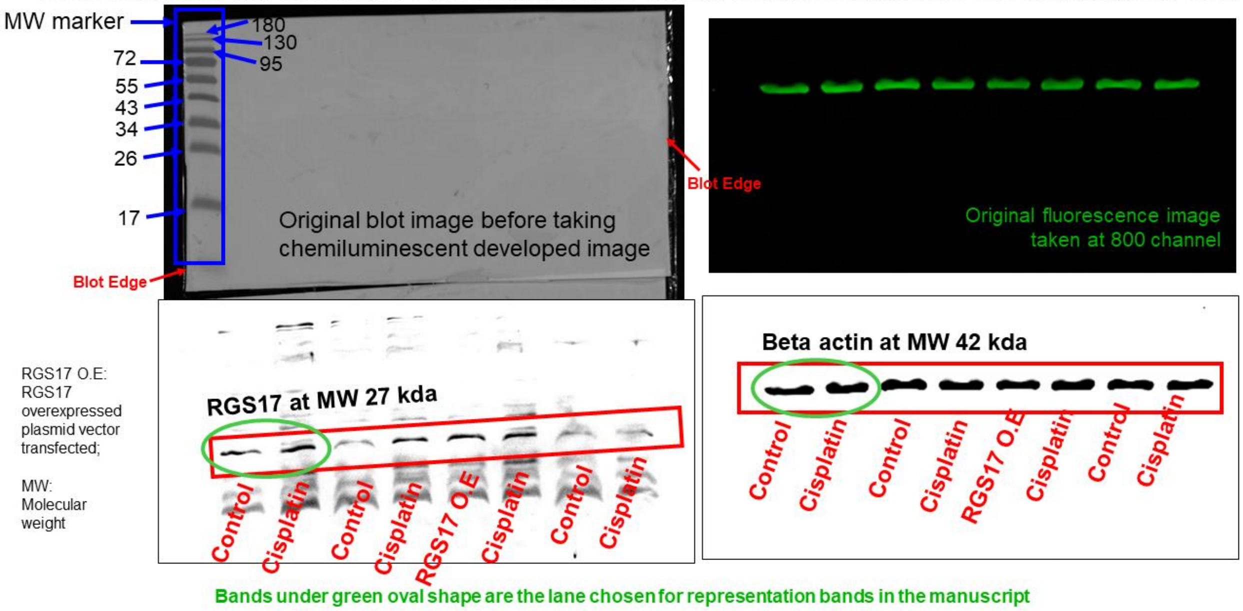

Bands under green oval shape are the lane chosen for representation bands in the manuscript

Figure 6A:

- Blot images were taken via Odyssey Imaging system by LI-COR biosciences
- Fluorescence western blotting technique was used to detect bands
  - pSTAT1 and STAT1 were detected at 700 channel
  - Beta actin was detected at 800 channel

For STAT1 blot

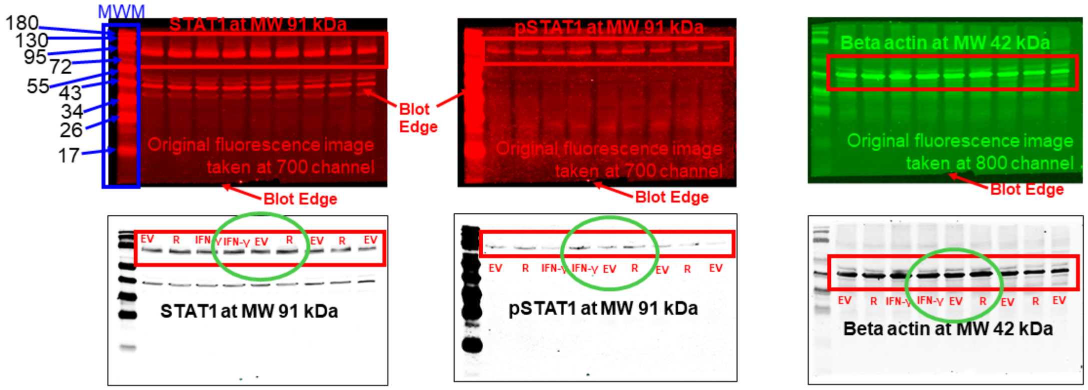

Bands under green oval shape are the lane chosen for representation bands in the manuscript

R: RGS17 overexpressed plasmid vector transfected; EV: Empty vector transfected MWM: Molecular weight marker

Figure 6A continued:

- Blot images were taken via Odyssey Imaging system by LI-COR biosciences
- Fluorescence western blotting technique was used to detect bands
  - pSTAT3, STAT3 and beta actin were detected at 800 channel

For STAT3 blot

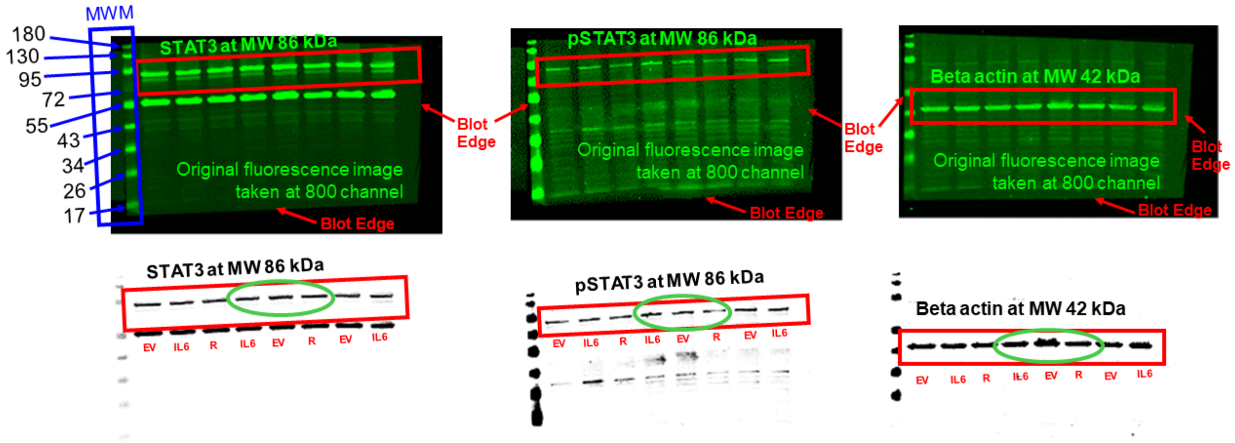

Bands under green oval shape are the lane chosen for representation bands in the manuscript

R: RGS17 overexpressed plasmid vector transfected; EV: Empty vector transfected MWM: Molecular weight marker

Supplementary fig 1A:

- RGS17 protein detected via Chemiluminescence western blotting technique
- EV: empty vector transfected UBOC1 cells
- RGS17 O.E: RGS17 overexpressed plasmid vector trasfected

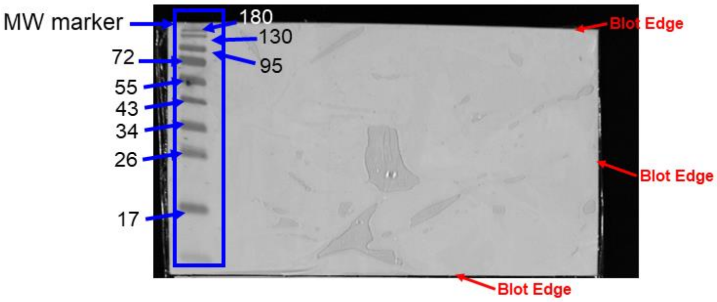

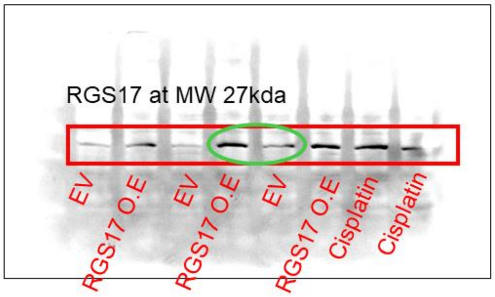

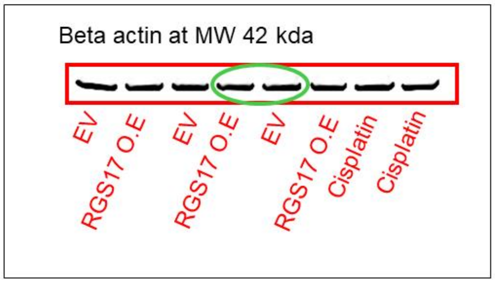

Bands under green oval shape are the lane chosen for representation bands in the manuscript

Supplementary Figure 5A:

- Blot images were taken via Odyssey Imaging system by LI-COR biosciences
- Fluorescence western blotting technique was used to detect bands
  - G alpha i (GNAI) were detected at 700 channel
  - Beta actin were detected at 800 channel

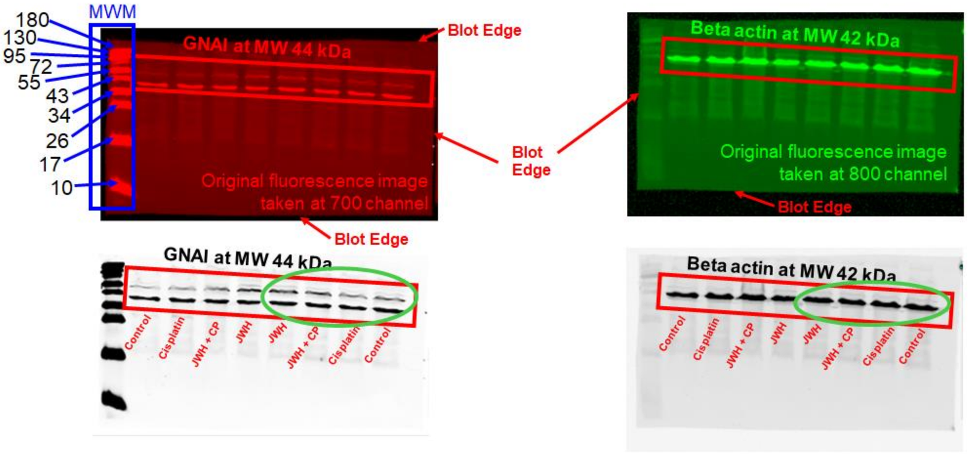

Bands under green oval shape are the lane chosen for representation bands in the manuscript

Cp: Cisplatin, MWM: Molecular weight marker

Supplementary Figure 5C:

- Blot images were taken via Odyssey Imaging system by LI-COR biosciences
- Fluorescence western blotting technique was used to detect bands
  - G alpha z (GNAZ) were detected at 700 channel
  - Beta actin were detected at 800 channel

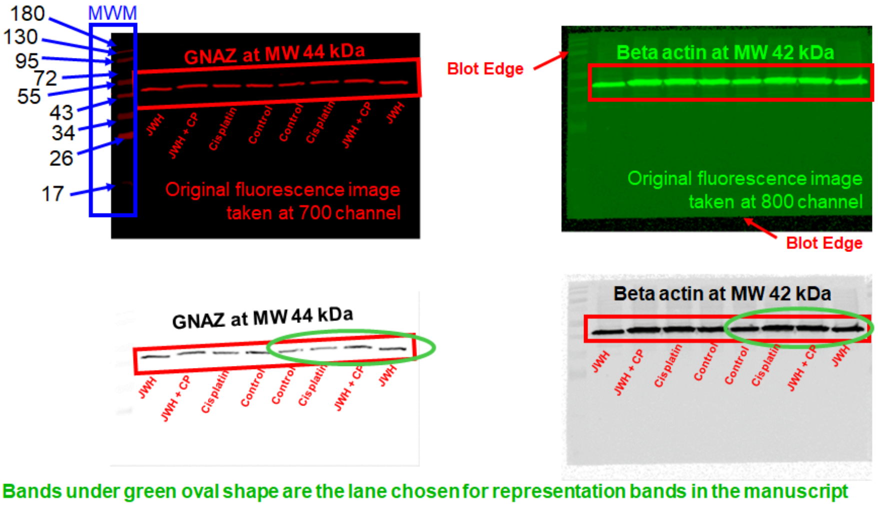

Bands under green oval shape are the lane chosen for representation bands in the manuscript

Cp: Cisplatin, MWM: Molecular weight marker; Predicted molecular weight of GNAZ 41kDa observed around 44.
